# Trajectory Simulation and Prediction of COVID‐19 *via* Compound Natural Factor (CNF) Model in EDBF Algorithm

**DOI:** 10.1029/2020EF001936

**Published:** 2021-04-05

**Authors:** Zhengkang Zuo, Sana Ullah, Lei Yan, Yiyuan Sun, Fei Peng, Kaiwen Jiang, Hongying Zhao

**Affiliations:** ^1^ Beijing Key Laboratory of Space Information Integration and 3s Application School of Earth and Space Science Peking University Beijing China; ^2^ School of Geosciences University of Edinburgh Edinburgh UK

## Abstract

Natural and non‐natural factors have combined effects on the trajectory of COVID‐19 pandemic, but it is difficult to make them separate. To address this problem, a two‐stepped methodology is proposed. First, a compound natural factor (CNF) model is developed via assigning weight to each of seven investigated natural factors, that is temperature, humidity, visibility, wind speed, barometric pressure, aerosol, and vegetation in order to show their coupling relationship with the COVID‐19 trajectory. Onward, the empirical distribution based framework (EDBF) is employed to iteratively optimize the coupling relationship between trajectory and CNF to express the real interaction. In addition, the collected data is considered from the backdate, that is about 23 days—which contains 14‐days incubation period and 9‐days invalid human response time—due to the nonavailability of prior information about the natural spreading of virus without any human intervention(s), and also lag effects of the weather change and social interventions on the observed trajectory due to the COVID‐19 incubation period; Second, the optimized CNF‐plus‐polynomial model is used to predict the future trajectory of COVID‐19. Results revealed that aerosol and visibility show the higher contribution to transmission, wind speed to death, and humidity followed by barometric pressure dominate the recovery rates, respectively. Consequently, the average effect of environmental change to COVID‐19 trajectory in China is minor in all variables, that is about −0.3%, +0.3%, and +0.1%, respectively. In this research, the response analysis of COVID‐19 trajectory to the compound natural interactions presents a new prospect on the part of global pandemic trajectory to environmental changes.

## Key Points

- The response of COVID‐19 trajectory to natural and non‐natural factors is separated through compound natural factor model
- Compound natural factor (CNF) exhibits the sensitive response to COVID‐19 trajectory
- Aerosol and visibility show the higher contribution to transmission, wind speed to death, and humidity followed by barometric pressure dominate the recovery rates, respectively
- Reduction in CNF value could help delay the spread of virus, but increase the death and decrease the recovery

## 1 Introduction

On March 11, 2020, the World Health Organization declared COVID‐19 a pandemic. Non‐pharmaceutical interventions (NPIs) helped China decrease a 67‐fold COVID‐19 cases (Lai, 2020), but still the possibility cannot be ruled out that the decrease is partially attributable to other unknown climatic factors. For example, temperature and absolute humidity. Many countries hope that the spread of COVID‐19 is likely constrained by climate, as the SARS in 2003. Some studies show temperature could have significant relationship to COVID‐19 transmission, and there might be an optimal temperature for the viral transmission (Wang and Jiang, et al., 2020; Wang and Tang, et al., 2020), and solar radiation threats the virus survival (Ahmadi, et al., 2020). However, some studies do not support the hypothesis that high temperature and UV radiation can be conductive in the reduction of COVID‐19 transmissibility. It might be premature to count on warmer weather to control COVID‐19 (Yao, et al., 2020; Zhu & Xie, 2020). Other climatic factors are also researched, such as humidity (Luo, et al., 2020; Ma, et al., 2020), aerosol (Wang & Du, 2020; Sima, 2020), wind speed (Ahmadi, et al., 2020; Islam, et al., 2020). Previous studies supported an epidemiological hypothesis that dry environments facilitate the survival and spread of droplet‐mediated viral diseases, and humid environments see attenuated viral transmission (Barreca & Shimshack, 2012; Shaman, et al., 2011). Next, reference (Ahmadi, et al., 2020; Wang and Tang, et al., 2020) show high humidity reduces the transmission of COVID‐19. However, reference (Luo, et al., 2020) concludes that the role of absolute humidity in transmission of COVID‐19 has not yet been established. In addition, COVID‐19 may transmit through aerosol (Liu, et al., 2020; Wang & Du, 2020), whereas there are also important reasons to suspect it plays a role in the high transmissibility of virus (Sima, 2020). Further, a study shows that an outbreak at low wind speed is remarkable (Islam, et al., 2020), but this result is nullified by another study (Oliveiros, et al., 2020). More contradictory conclusions about the reported climatic influences on the pandemic were shown in Table S5. Correspondingly, our research will be, to some extent, a trade‐off for those contradictory conclusions, because we will combine all investigated parameters into a single compound model to consider their coupling relationship for more reasonable and logical conclusions about the climatic influence on the COVID‐19 pandemic.

However, why these studies show diverged results? It is still not clear that how climate play its part in the transmissibility of COVID‐19. The spreading mechanism of virus is very complex, coupling certain factors. NATURE and SCIENCE published articles affirming the positive effect of pre‐emptive implementation of NPIs on mitigating the pandemic (Lai, 2020; Tian, et al., 2020). Considering the impact of natural factors on virus transmission along and excluding the NPIs, it is still insignificant method to do independent analysis on the part of considering single natural factors (SNFs) and to ignore their coupling relationship (CR). However, the CR received less attention in the COVID‐19 modeling communities. One of the potential solutions is weighted ensemble method, which is popular in Meteorology (Yoo, et al., 2020), Socioeconomics (Boyce, et al., 2020), and Climatology (Strobach, et al., 2020). Therefore, the primary objective of this study was to quantify the influence of compound natural factor (CNF) on COVID‐19 trajectory, and to quantify the contributions of their potential driving factors, including temperature, humidity, visibility, barometric pressure, wind speed, aerosol, and vegetation. We used the mean monthly case growth rate (Tellis, et al., 2020), death growth rate (Ma, et al., 2020), and recovery growth rate during 3 months (January, February, March) in 31 Chinese cities as proxies for COVID‐19 trajectory. Seven mean monthly natural factors during the same timestamp were used to quantify the environmental changes in 31 Chinese cities. We also analyzed the relative contribution of each natural factor to COVID‐19 trajectory from January 22 to February 12, 2020. To analyze the observed changes in trajectory during March, we designed three CNF models (M_1_: infection; M_2_: death; M_3_: recovery) to predict the contemporaneous trajectory driven by environmental changes. For comparison to the traditional single natural factor analysis method, we used an empirical distribution based framework (EDBF) (Zhu et al., 2020) to minimize model uncertainties by optimizing the integration of model simulations. Onward, the influence of natural and non‐natural factors is potentially separated through the deviation of predicted trajectory from observed trajectory.

## 2 Materials and Methods

### 2.1 Data Collection

In this study, for experimental purpose 31 Chinese cities, that is 27 provincial capitals and 4 metropolitan cities (Beijing, Shanghai, Tianjin, and Chongqing) are considered, wherein 6 city‐wise pandemic parameters data and 7 single natural factors (SNFs) data from January 22, 2020 to March 18, 2020 are collected. Moreover, the city‐wise pandemic data including new/cumulative cases per day, new/cumulative deaths per day, and new/cumulative recoveries per day is collected from the Pandemic Real‐time Reports on the website of 31 Provincial Health Commission of People's Republic of China (Table S1). In addition, SNFs include meteorological data, that is temperature, humidity, visibility, wind speed, and barometric pressure, which is collected from the weather data repository (https://weatherspark.com/). Besides, the aerosol optical depth (AOD) data, and fractional vegetation coverage (FVC) data are collected from the National Aeronautics and Space Administration (NASA) (https://giovanni.gsfc.nasa.gov/giovanni) and National Earth System Science Data Center (http://www.geodata.cn/index.html), respectively. Finally, all data for this study are available in the CNF‐Model data repository (https://github.com/ZhengkangZUO-2020/CNF-Model).

### 2.2 Pandemic Metric

Daily growth rate is one of the simple, intuitive, and generalizable metrics to interpret the spread of COVID‐19 pandemic (Tellis, et al., 2020). Daily growth rate is the percentage increase in cumulative cases, deaths, and recoveries, which is not dependent on calendar time, country, or type of disease. Therefore, this feature enables comparison across time and country. For example, when Wuhan reported 356 new cases on January 29 on a base of 1,905 total cases on January 28, its case growth rate (CGR) was 19%. At that rate, the number of victims would have grown to about 4,546 in 5 days. Had Governor not intervened and allowed the disease to spread uncontrolled, the disease would have infected 498,000 victims as of February 29. Similarly, death growth rate (DGR) and recovery growth rate (RGR) can be also calculated as Tellis mentioned. Using this metric, Tellis also defined three measurable benchmarks for analysts and public managers to target: when case growth rate stays below 10%, 1%, and 0.1%, the pandemic is defined as moderation, control, and containment, respectively.

### 2.3 EDBF Optimizer

The Compound Natural Factor (CNF) model applied in this study is mainly based on our previous work presented in (Ullah, et al., 2020; Zhu et al., 2020), where the basis function, that is Equation 1 is selected at an optimum weight and by interpolating the correlation between weighted natural factors and pandemic variables. After successfully applying the proposed methodology, the Empirical Distribution based Framework (EDBF) (Zhu et al., 2020) was employed in optimizing the CNF model. From the EDBF algorithm perspective, it is a general framework rather than a specific algorithm, which is easy to implement and can easily accommodate any existing multi‐parent crossover algorithms (MCAs). Moreover, the existing MCA‐based coefficients (Eiben & Bäck, 1997; Goldberg, 1991; Herrera, et al., 1998) follow a uniform distribution, which also violates constraints, thus propagate error. Errors cascade exponentially, with even a slight increase in the hybrid scale, which leads to the increase in time consumption. To address such problem, EDBF is the best solution which takes multiple MCAs as its constituent members. In addition, the number of iterations during the execution of EDBF algorithm was set to 50,000 with the reason that a possible number of iterations be available for the stabilization of convergence before the ending of simulation process. Though the convergence stabilized before a 50,000 number of iterations, still a slight improvement could be observed, and further improvement in the regression value(s) could be expected. Instead, by terminating simulation during the execution, we let simulation process to be completed until the last iteration. Moreover, the parameters setting in EDBF algorithm to optimize CNF model is 200‐sized population pool, 15 parent chromosomes with 5 elitists, and each chromosome incorporates seven genes (weight of each SNFs).

### 2.4 CNF Model

COVID‐19 trajectories are the result of the combined actions of multiple natural factors, and each factor has different influence. In this regard, separate weight value should be assigned to the single natural factors (SNFs) on the basis of its influence. Based on weighted SNFs outputs, the most influencing nature predictor that predicts the impending COVID‐19 trajectory is considered for further evaluation through EDBF algorithm. In this research, the developed methodology is based on the earlier work of (Ullah, et al., 2020; Zhu et al., 2020). The framework of CNF model is shown in Figure S1. Based on calculated *r* values, the process starts through randomly generating initial weight vector *W*, which by substituting into Equation 2 obtains CNF:

(1) $$\text{CNF}={w_{T}}^{\prime }T+{w_{H}}^{\prime }H+{w_{V}}^{\prime }V+{w_{B}}^{\prime }B+{w_{W}}^{\prime }W+{w_{A}}^{\prime }A+{w_{F}}^{\prime }F$$

where CNF is the weighted natural factor, $W=\left\{w_{T}{\text{,w}}_{H}{\text{,w}}_{V}{\text{,w}}_{B}{\text{,w}}_{W}{\text{,w}}_{A}{\text{,w}}_{F}\right\}$ corresponds to the weight values (Eq. [2]), and vector *T*, *H*, *V*, *B*, *W*, *A* and *F* corresponds to each of the seven natural factors, that is temperature, humidity, visibility, barometric pressure, wind speed, aerosol, and vegetation, respectively.

(2) $$w_{T}+w_{H}+w_{V}+w_{B}+w_{W}+w_{A}+w_{F}=1$$

Subsequently, the correlation coefficient R_CNF‐C_, R_CNF‐D_, and R_CNF‐R_ between CNF and each pandemic variable is calculated, respectively. In addition, EDBF algorithm is run to iteratively optimize *W* to obtain and accurate weight vector *W*_*t*_, where *t* represents the number of iterations. Moreover, relationships between CNF and pandemic predictors are evaluated, respectively. Hereafter, the optimal weight vector of CNF model is used in the prediction of COVID‐19 trajectory. Finally, the observed trajectory in March is compared with the predicted one to measure the model accuracy.

### 2.5 Model Evaluation Metric

Taylor diagram is useful in evaluating multiple aspects of models (Taylor, 2001), which characterizes the statistical relationship between two fields, a “test” field (often representing a field simulated by a model) and a “reference” field (usually representing “truth,” based on observation). The similarity between two fields is quantified in terms of their correlation, their centered root‐mean‐square difference and their standard deviation. The reason that each point in the two‐dimensional space of the Taylor diagram can represent three different statistics simultaneously is that these statistics are related by the follow formula:

(3) $$E\prime^{2}={\sigma_{f}}^{2}+{\sigma_{r}}^{2}-2\sigma_{f}\sigma_{r}R$$

where *R* is the correlation coefficient between the test and reference field, E′ is the centered RMS difference between the fields, and ${\sigma_{f}}^{2}$ and ${\sigma_{r}}^{2}$ are the variances of the test and reference fields, respectively.

### 2.6 Lag Effect Compensation

**Figure 1:**
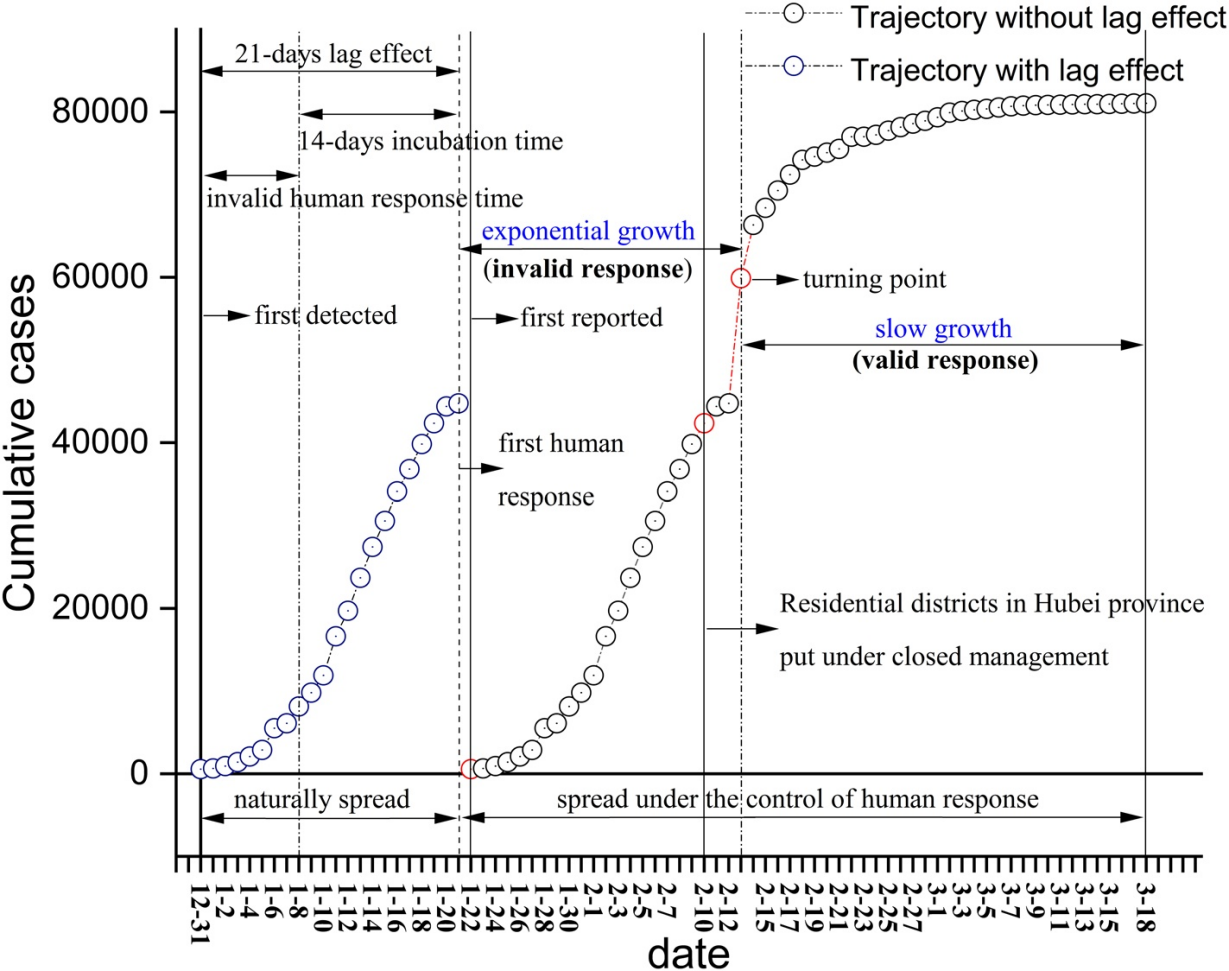
The characteristic of COVID‐19 pandemic trajectory between December 31, 2019 and March 18, 2020 in China. Respiratory disease due to novel coronavirus detected in Wuhan city on December 31, 2019, Ministry of Transport launches Level 2 emergency on January 21, 2020, Health Commission of People's Republic of China (HCPRC) reported the first epidemiological data on January 22, Residential districts in Hubei province put under closed management on February 10, the first turning point emerged on February 13, and the COVID‐19 pandemic began retreat back on March 18. The virus spread naturally without any human interventions between December 31, 2019 and January 21, 2020. Onward, the virus went on spreading under the control of human response between January 22 and March 18. Specifically, the COVID‐19 pandemic exhibited the exponential growth between January 22 and February 13, while had the slow growth between February 14 and March 18.

The obstacles of revealing the more real interaction between the COVID‐19 trajectory and compound natural factor are: 1) no official epidemiological data where the virus naturally spread without any human interventions; 2) lag effects of weather change and social intervention on the observed trajectory of COVID‐19 due to the incubation period of COVID‐19. In Figure 1, the COVID‐19 virus exhibited 23‐days exponential growth (between January 22 and February 23) and 33‐days slow growth (between February 24 and March 18). It is noteworthy to mention that the turning point of COVID‐19 trajectory emerged on February 23. Therefore, there are certainly invalid time for human response to the overwhelming attack from the virus, especially at the beginning of the outbreak. We assumed 9 days as the average invalid human response time, which would cause 9‐days lag effect to the COVID‐19 trajectory. Combined with 14‐days incubation period of COVID‐19 (Lauer, et al., 2020), there are 23‐days lag effect requisite to be compensated.

To handle these two problems, the reported data was considered from the backdate, that is about 23 days earlier which contains 14‐days COVID‐19 incubation period and average 9‐days invalid human response time. Based on this assumption, the reported data—between January 22 and February 12, 2020—approximately reveals the non‐reported COVID‐19 trajectory from the backdate—between December 31, 2019 and January 21, 2020—during which the virus naturally spread (Figure 1).

## 3 Results

### 3.1 Single Natural Factors Acting on COVID‐19 Trajectory

**Figure 2:**
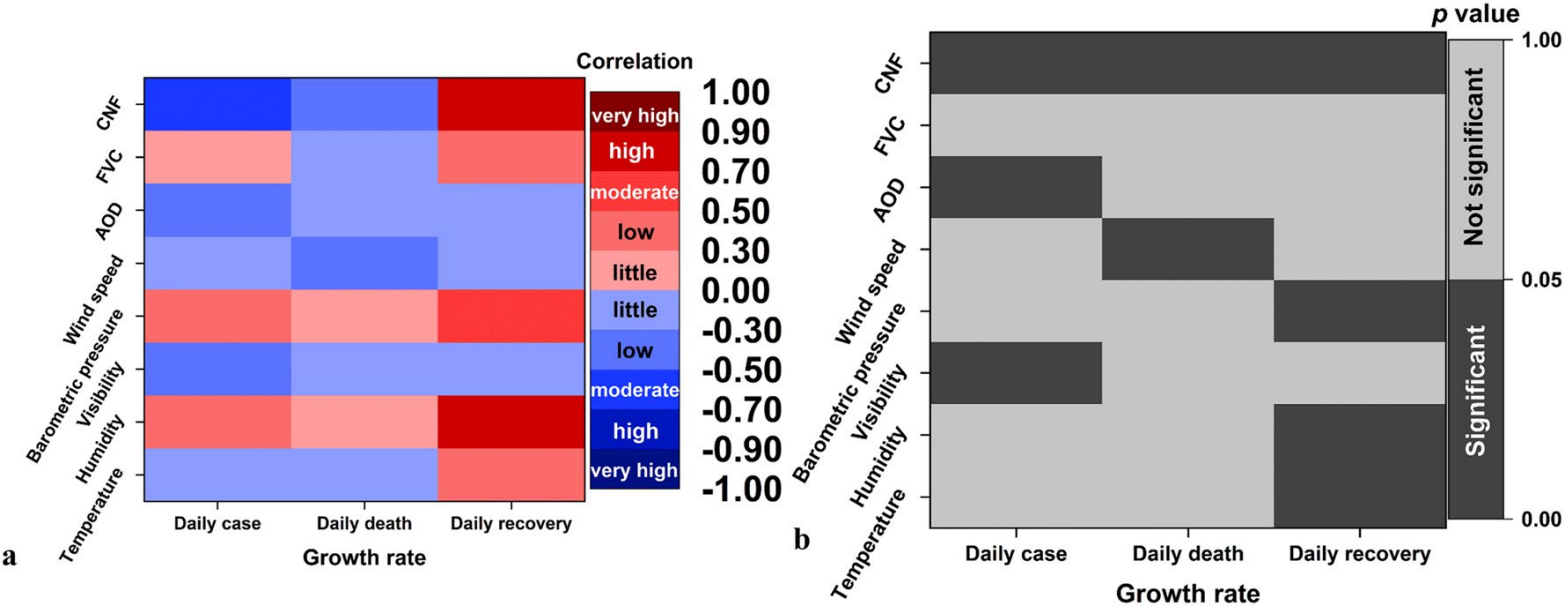
Correlation and significance of natural factors acting on COVID‐19 trajectory. The left diagram represents the correlation matrix between SNFs and pandemic variables, where red colors and blue colors indicate the positive and negative influence, respectively. Correlation coefficients whose magnitude are between 0.7 and 0.9 indicate variables which can be considered highly correlated, while moderate correlation exists between 0.5 and 0.7, low correlation (0.3–0.5) and little correlation (0–0.3), respectively. The right diagram represents the corresponding p value matrix, where black colors and gray colors individually indicate the statistically significant (*p* < 0.05) and insignificant (*p* > 0.05) correlation between natural factors and pandemic variables.

The execution of proposed CNF model was first formulated through evaluating the COVID‐19 trajectory response, for example, each pandemic variable with respect to the seven single natural factors (SNFs). Additionally, each investigated pandemic variable, for example, growth rate in terms of case, death and recovery was plotted against each SNFs. Demonstration through scatter diagrams and polynomial regression (Figure S3) described the relationship between pandemic variable and SNFs, that is temperature, humidity, visibility, barometric pressure, wind speed, aerosol and vegetation, respectively. Moreover, the influence of natural factors on COVID‐19 trajectory is illustrated in Figure 2 through *r* and *p* values (Table S2). It was observed that only two SNFs, that is aerosol and visibility have a statistically significant response to the case growth rate at the 0.01 and 0.05 significance level, showing a moderate negative response (*r* = −0.457, *p* = 0.01) and low negative response (*r* = −0.399, *p* = 0.026), respectively. In addition, the unique significant relationship between SNFs and the death growth rate was observed at wind speed, showing a low negative response (*r* = −0.365, *p* = 0.043). Furthermore, another three SNFs, that is humidity, barometric pressure and temperature have a significant response to the recovery growth rate, showing a high positive response (*r* = 0.724, *p* < 0.001), moderate positive response (*r* = 0.671, *p* < 0.001) and low positive response (*r* = 0.414, *p* = 0.021), respectively. It was mentioned that only vegetation has no significant response to all three pandemic variables. On the contrary, compound natural factor (CNF) had significant response to all pandemic variables, showing a stronger response than all SNFs. Notably, the details of CNF results could be found in Section 3.2.

### 3.2 Coupling Relationship in Weighted Natural Factor

**Figure 3:**
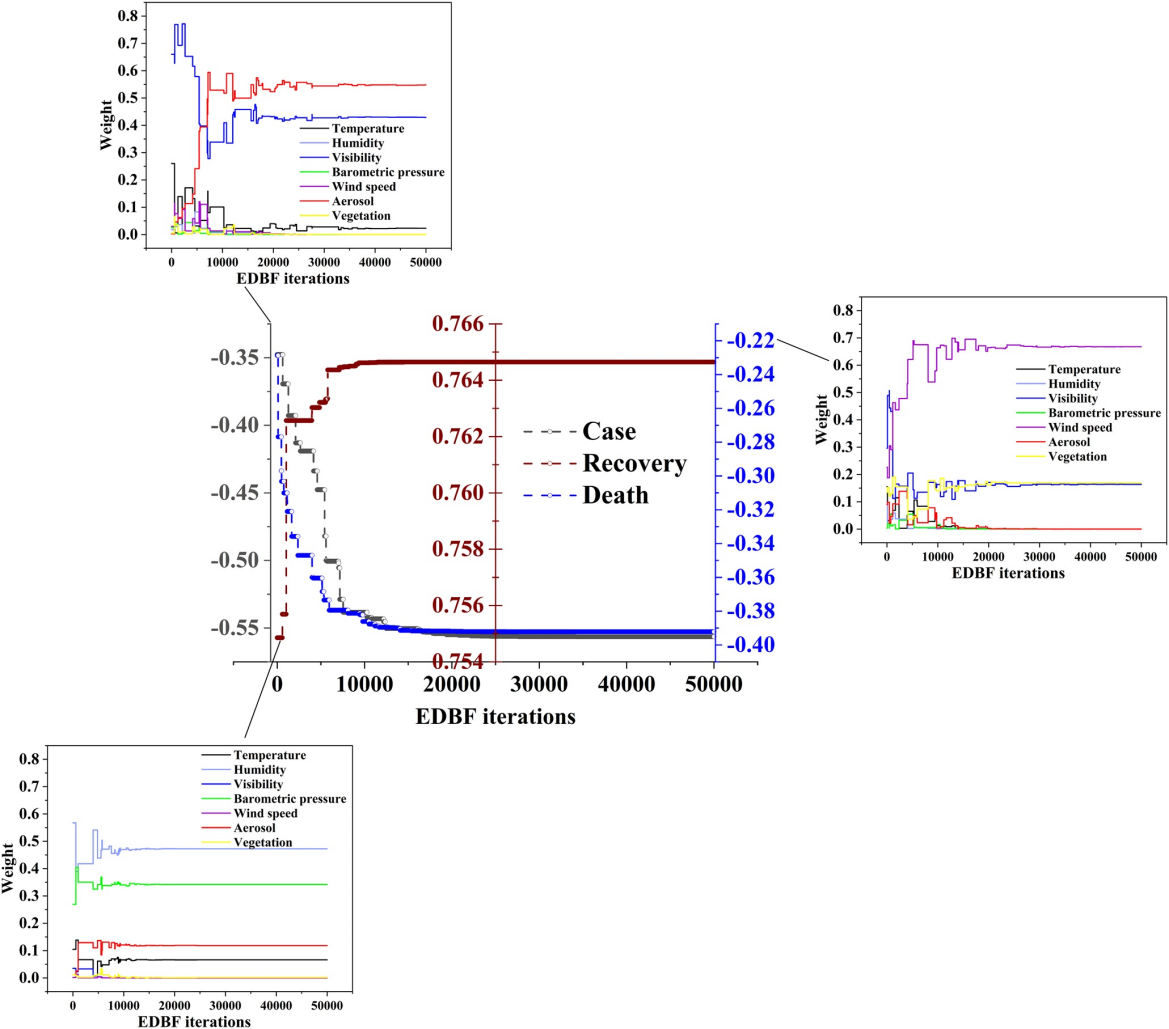
The optimization process of weights in CNF model at each pandemic variables, such as growth rate in terms of case, death and recovery. The middle diagram represents the evolutionary *r* values between CNF model outputs and pandemic variables. The left‐top, the right and the left‐down diagrams illustrate the weight assigned to each SNFs was optimizing with the increase of EDBF iterations for improving *r* values between CNF model outputs and growth rate in terms of case, death and recovery, respectively.

In the EDBF algorithm, initial weight values were randomly assigned to each single natural factor, respectively (Figure S2). Onward, the optimal weight values were evaluated through EDBF algorithm, and the number of iterations was set to 50,000. Figure 3 demonstrates the iteration wise statistics at each pandemic variable, in which figures at the location of top, right, and bottom show weight values and the figure at the location of middle shows *r* values, which were iteratively generated by the algorithm itself. To investigate weight values, it was observed that lots of discrepancies exist in the convergence of investigated variables, and the convergence showed stabilization onward 40,000 iterations at case growth rate (Figure 3‐top), 35,000 iterations at death growth rate (Figure 3‐right) and 15,000 iterations at recovery growth rate (Figure 3‐down). In Figure 3‐top, the aerosol; Figure 3‐right, wind speed, and vegetation; Figure 3‐down, barometric pressure and aerosol, respectively, showed higher weight value from the beginning until the last iteration. As for *r* values are concerned, uncertainty in initial iterations was observed as shown in Figure 3‐middle, and the convergence showed stabilization onward 15,000 iterations. Likewise, it was also observed that the absolute *r* values drastically increased before the stabilization of convergence.

Furthermore, the optimal weight values in CNF model at each pandemic variable were shown in Figure S2, wherein it showed that the aerosol (54.8%) followed by the visibility (42.9%) and the temperature (2.3%) are the most influencing SNFs on the coupling relationship in the CNF model which is constructed on the data with respect to COVID‐19 case growth rate. However, the humidity, the barometric pressure, the wind speed and the vegetation do not contribute to the CNF model. As far the CNF model—constructed on the death growth rate—are concerned, the wind speed (66.8%) followed by the vegetation (16.9%) and the visibility (16.4%) had higher impacts. Onward, in the CNF model about recovery growth rate, the weight of humidity is 47.2%, barometric pressure (34.2%), aerosol (11.8%), temperature (6.6%), vegetation (0.1%), wind speed, and visibility have no contribution. In addition, the weighted *r* value predicted by EDBF algorithm was higher as compared to the calculated *r* value for each single natural factors at each pandemic variable, as shown in Figure 3. The highest weighted *r* was predicted at recovery growth rate (0.765) followed by case growth rate (−0.556) and death growth rate (−0.392), respectively.

### 3.3 CNF‐Model Evaluation

**Figure 4:**
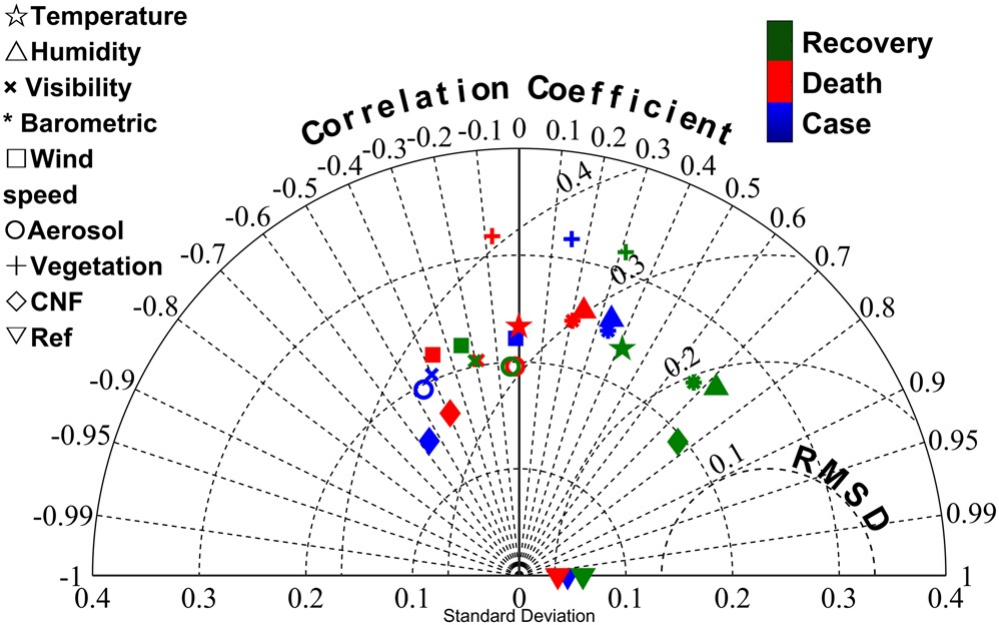
Taylor diagram (Taylor, 2001) displaying a statistical comparison with observations of seven SNFs and CNF model estimates of the COVID‐19 pandemic trajectories, wherein 24 models are divided into three groups based on the pandemic variables, and each group has eight models which is separated by natural factors. It is mentioned that the blue, the red, and the green markers represent the case growth rate, the death growth rate and the recovery growth rate, respectively. Onward, in each group, the pentagram, the upper triangle, the cross, the asterisk, the square, the circle, the plus, the rhombic, and lower triangle marks represent the temperature, the humidity, the visibility, the barometric pressure, the wind speed, the aerosol, the vegetation, the compound natural factor, and the observed data, respectively. The standard deviation shows the variability of the observed and the modeled COVID‐19 trajectory. The distance of points to the matched lower triangle on the *x*‐axis identified as “Ref” show centered root mean square difference (RMSD) between model simulations and observation.

We evaluated the performance of the CNF model in simulating COVID‐19 trajectory in China using the Taylor diagram (Figure 4). Trajectory simulated by CNF model and seven SNF models were compared to observed trajectory. The performance of the modeled trajectory was quantified by correlation coefficients (*R*) between the modeled and observed trajectory, standard deviation (SD) of the variation in the spatial trajectory, and the root mean square difference (RMSD) between the modeled and observed trajectory. For the study area, absolute correlation coefficients between model‐simulated trajectory and observed trajectory ranged from $0.186{\;}_{0.002,\;\text{temperature}}^{0.392,\;\text{CNF}}$($\text{mean}{\;}_{\text{min}}^{\text{max}}$) for Death, to $0.285{\;}_{0.015,\;\text{wind}\;\text{speed}}^{0.556,\;\text{CNF}}$ for Case, to $0.422{\;}_{0.041,\;\text{aerosol}}^{0.765,\;\text{CNF}}$ for Recovery. SD and RMSD values between the modeled and observed trajectory also suggest overall acceptable performance by the CNF models in reproducing observed spatial trajectory variation (SD ranging from $3.909{\;}_{3.253,\;\text{CNF}}^{5.336,\;\text{vegetation}}$ for Recovery, to $5.029{\;}_{3.339,\;\text{CNF}}^{7.026,\;\text{vegetation}}$ for Case, to $6.355{\;}_{4.563,\;\text{CNF}}^{8.814,\;\text{vegetation}}$ for Death, and RMSD ranging from $0.051{\;}_{0.024,\;\text{CNF}}^{0.093\text{,}\;\text{vegetation}}$ for Recovery, to $0.057{\;}_{0.033,\;\text{CNF}}^{0.105,\;\text{vegetation}}$ for Death, to $0.057{\;}_{0.033,\;\text{CNF}}^{0.099,\;\text{vegetation}}$ for Case). Onward, it is mentioned in Equation 3 that $E^{\prime }$ will be less with larger *R* and less difference of $\sigma_{f}$ from $\sigma_{r}$. Thus, less $E^{\prime }$ reveals higher accuracy of simulated model because of the closer distance from the observed. In this regard, CNF model illustrated the highest accuracy than SNF models at each pandemic variable (Figure S4).

### 3.4 CNF‐Based Prediction of COVID‐19 Transmission

The polynomial model developed with case growth rate (CGR) and compound natural factor (CNF) during the invalid human response time (COVID‐19 virus spread naturally, which will be discussed in Section 4.2) is shown in Equation 4. Due to the hypothesis that the delayed response of COVID‐19 trajectory to human intervention, 22‐days (1–22 to 2–12) of reported trajectory essentially revealed preexistent trajectory 22 days ago, that is during December 31, 2019 and January 21, 2020. That is to say, reported trajectory always exists the hysteretic nature (i.e., assumed 22 days based on simple analysis in Section 4.2) especially at the beginning of the COVID‐19 outbreak. We have to employ existing reported data to approximately simulate the natural spread of COVID‐19 virus because there are no reported data available before January 22, 2020 (the first human response time, before which virus spread naturally). In this regard, Equation 4 in conjunction with Equation 1 revealed the interaction between natural COVID‐19 transmission and compound natural factor which is weighed by temperature, humidity, visibility, wind speed, barometric pressure, aerosol, and vegetation.

(4) $$\text{CGR}\left(\text{CNF}\right)=-1.79{\text{CNF}}^{3}+2.172{\text{CNF}}^{2}-0.8749\text{CNF}+0.2309$$

**Figure 5:**
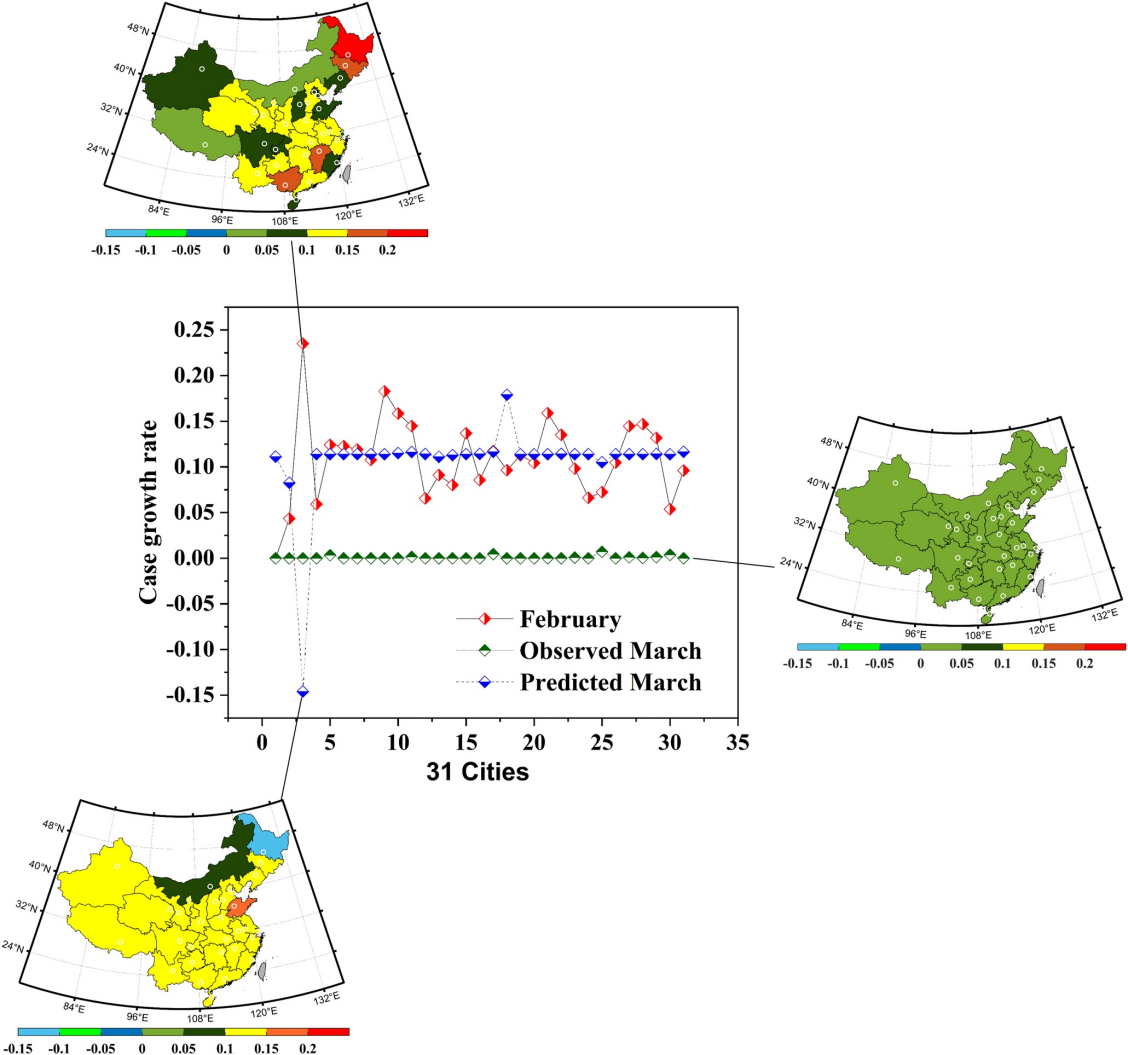
CNF‐based prediction of case growth rate during valid human response time to COVID‐19. The middle diagram represents the observed monthly shift of COVID‐19 transmission from February (red line) to March (green line), and the deviation between the predicted (blue line) and observed transmission. The left‐top, the right, and the left‐down diagrams illustrate the spatial distribution of COVID‐19 related case during February, March, and predicted March, respectively.

Onward, demonstration through scatter diagrams and polynomial regression of Equation 4 is shown in Figure S3i−S3h. Furthermore, seven single natural factors during valid human response time (2–13 to 3–18) is substituted into Equation 1, as the recombine of the contemporaneous compound natural factor. It is worthy to mention in Equation 1 that the weight assigned to each single natural factor is the equivalent of individual responsive strength to COVID‐19 trajectory. Subsequently, recombined compound natural factor is substituted into Equation 4, the output of which revealed the natural transmission of COVID‐19 virus and interacted mechanism of 7 investigated natural factors. The predicted trajectory is shown in Figure 5. The spatial COVID‐19 pattern of 31 cities in China during invalid human response time is quite dispersive (Figure 5‐top), wherein Harbin has 23.5% of average daily growth rate in case, followed by Nanning (18.3%), Nanchang (15.9%), Changchun (15.9%), Zhengzhou (14.7%), Guiyang (14.5%), Wuhan (14.5%), etc., There are 18 investigated cities (over 58%) stayed upper 10% of case growth rate. Based on Section 2.2, case growth rate staying below 10%, 1%, and 0.1%, the pandemic is defined as moderation, control, and containment, respectively. Thus during the virus natural transmission period, pandemic in 12 investigated cities (38.7%) was defined as moderation, only Lhasa was the containment, while 18 cities were out of control.

However, supposing that the virus spread naturally in subsequent 47 days (2–13 to 3–18), the predicted COVID‐19 transmission is shown in Figure 5‐down. Obviously, the spatial COVID‐19 pattern became uniform with the interaction of compound natural factor. Specifically, the variety of predicted case growth rate in Harbin seems outlier (23.5% to −14.6%), that is because the shift of compound natural factor in Harbin is enormous (5.4% to 93.1%) (Figure S5a). It is noteworthy that there is similarly uniform spatial COVID‐19 pattern between the observed pattern (Figure 5‐right) and the contemporaneous predicted pattern. This phenomenon and its further separation of influence between natural and non‐natural factors to COVID‐19 trajectory will be discussed in Section 4.3.

### 3.5 CNF‐Based Prediction of COVID‐19 Related Death

The polynomial model developed with death growth rate (DGR) and compound natural factor (CNF) during the invalid human response time (COVID‐19 virus spread naturally) is shown in Equation 5 and Figure S3‐II‐h.

(5) $$\text{DGR}\left(\text{CNF}\right)=-1.019{\text{CNF}}^{3}+1.625{\text{CNF}}^{2}-0.8444\text{CNF}+0.1534$$

**Figure 6:**
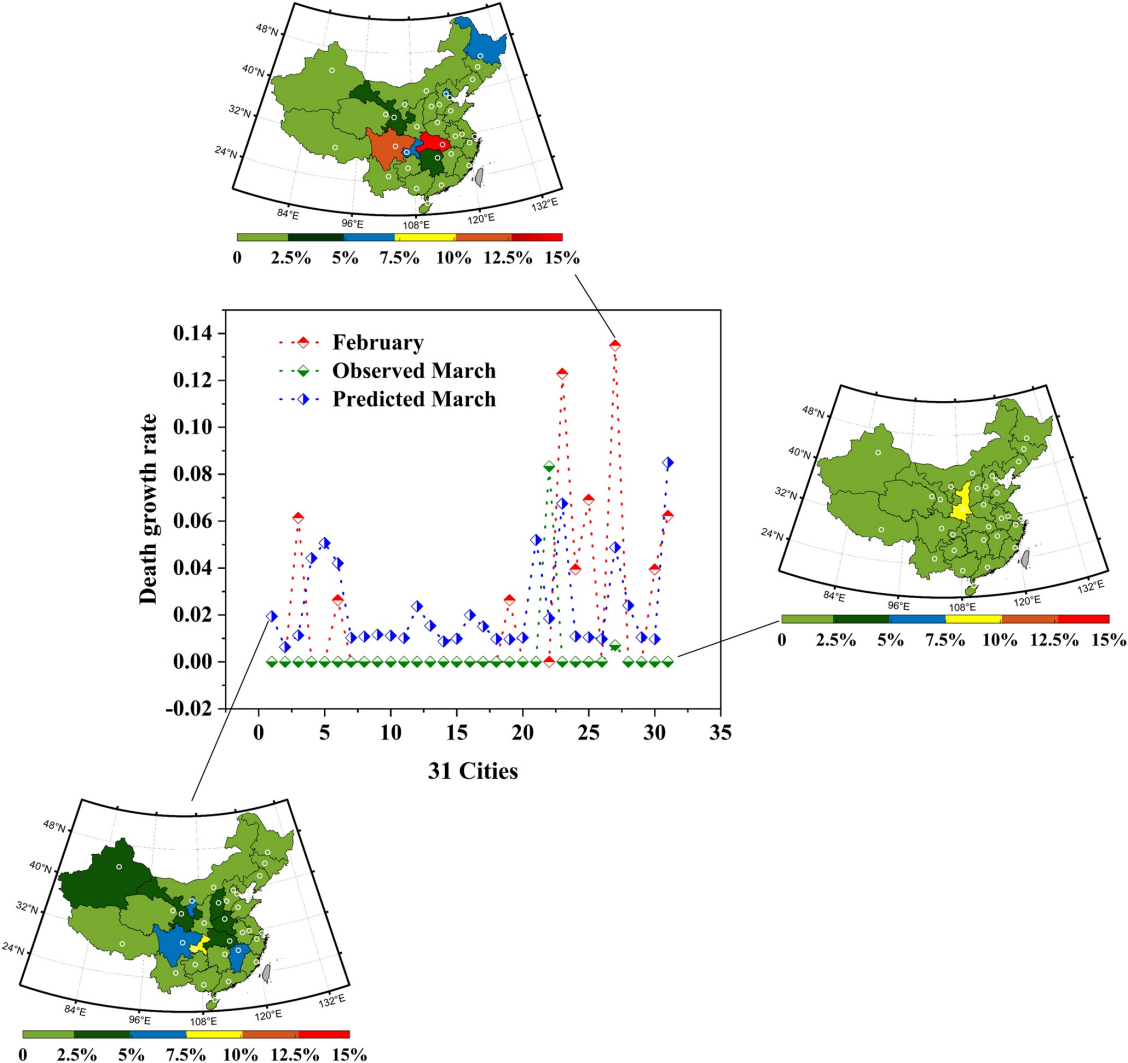
CNF‐based prediction of death growth rate during valid human response time to COVID‐19. The middle diagram represents the observed monthly shift of COVID‐19 related death from February (red line) to March (green line), and the deviation between the predicted (blue line) and observed transmission. The left‐top, the right and the left‐down diagrams illustrate the spatial distribution of COVID‐19 related death during February, March, and predicted March, respectively.

As the similar process in Section 3.3, during the valid human response time (Table S3), the contemporaneous recombined compound natural factor is simulated through the substitution of seven single natural factors into Equation 1. Subsequently, recombined compound natural factor is substituted into Equation 5, the output of which revealed the natural COVID‐19 trajectory concerning death and interacted mechanism of 7 investigated natural factors. The predicted trajectory is shown in Figure 6. During the invalid human response time to COVID‐19 trajectory, nine cities (29%) exhibited the notable average daily death growth rate (over 10%), wherein Wuhan (13.5%), followed by Chengdu (12.3%), Beijing (6.9%), Chongqing (6.2%), Harbin (6.1%), and Shanghai (3.9%), etc., However, daily death growth rate in other 22 cities (71%) is at the very low level (Figure 6‐top). Onward, during the valid human response time, except for Xian, 30 cities exhibited the decrease in death growth rate (Figure 6‐right). However, supposing that there is no valid human response to COVID‐19, the predicted trajectory concerning death is shown in Figure 6‐down. Onward, the simulated COVID‐19 trajectory concerning death was region specific due to the environmental change variety, wherein only seven cities exhibited the decrease trend, while 24 cities showed the increase trend. It is mentioned that Wuhan decreased 8.6%, followed by Beijing (−5.9%), Chengdu (−5.5%), Harbin (−5%), Shanghai (−3%), Tianjin (−2.9%), and Changsha (−1.7%). On the contrary, Nanchang increased prominently (+5.2%), followed by Yinchuan (+5.1%), and Urumqi (+4.4%), etc.,

### 3.6 CNF‐Based Prediction of COVID‐19 Related Recovery

The polynomial model developed with recovery growth rate (RGR) and compound natural factor (CNF) during the invalid human response time (COVID‐19 virus spread naturally) is shown in Equation 6 and Figure S3‐III‐h.

(6) RGR(CNF)=0.235CNF−0.02306

**Figure 7:**
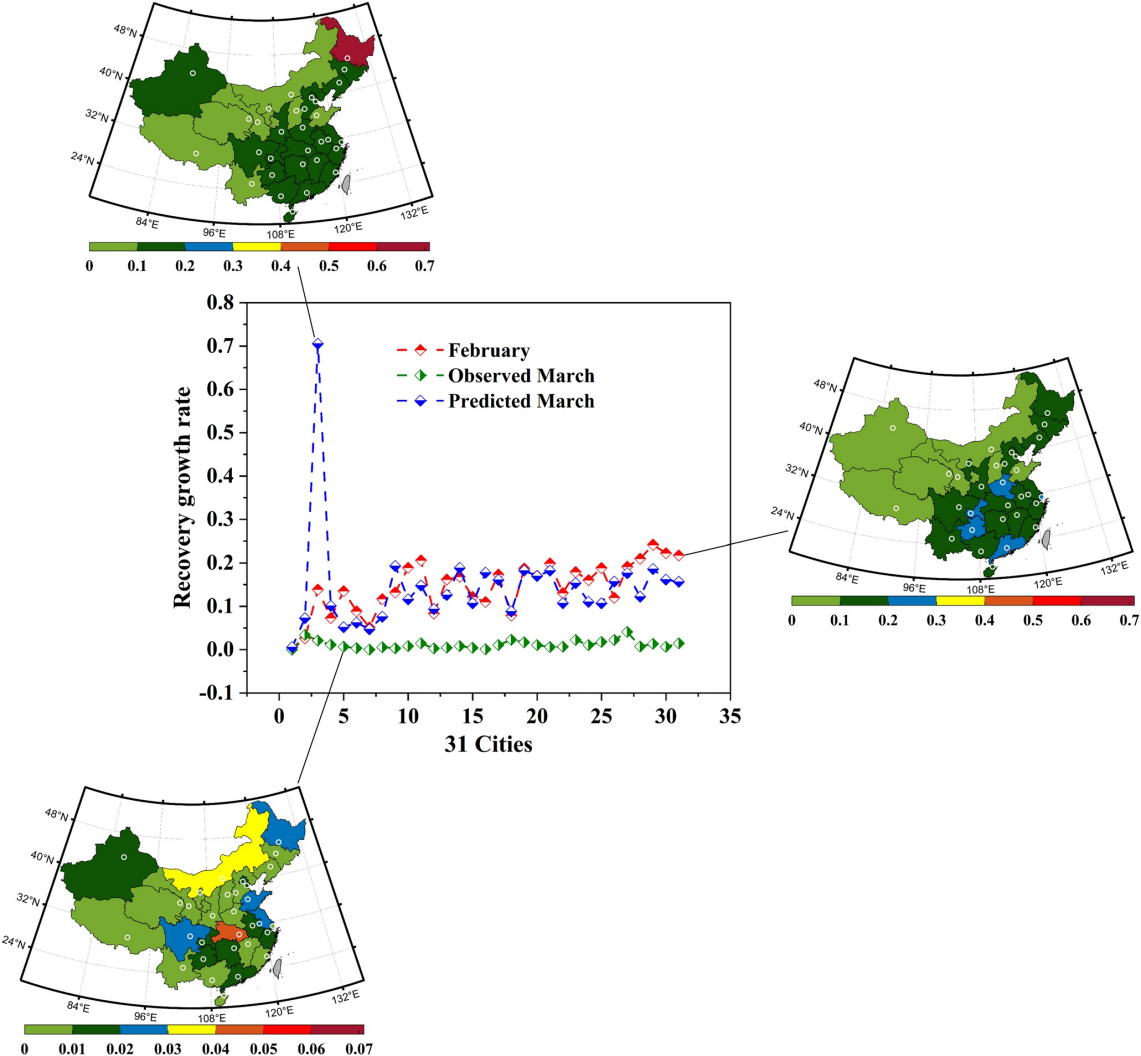
CNF‐based prediction of recovery growth rate during valid human response time to COVID‐19. The middle diagram represents the observed monthly shift of COVID‐19 related recovery from February (red line) to March (green line), and the deviation between the predicted (blue line) and observed transmission. The left‐top, the right and the left‐down diagrams illustrate the spatial distribution of COVID‐19 related recovery during February, March, and predicted March, respectively.

As previous mention in Section 3.3, the COVID‐19 virus spread naturally during the invalid human response time (Table S3). Similarly, supposing that the natural response still dominates the virus spread during the valid human response time (Table S3), COVID‐19 trajectory concerning recovery (Figure 7‐down) is predicted through Equation 1 and Equation 6. It is mentioned in Figure 7‐top that, during the invalid human response time, 5 cities exhibited over 20% of average daily recovery growth rate, while 18 cities over 10% and seven cities under 10%. Specifically, in the notable daily recovery growth rate, Guangdong exhibited 24.2%, followed by Shanghai (22.3%), Chongqing (21.8%), Zhengzhou (21%), and Guiyang (20.6%). Besides, for the growth rate at the moderate level, Nanchang exhibited 19.9%, followed by Wuhan (19.1%), Changchun (18.9%), and Beijing (18.9%), etc., Furthermore, Lhasa exhibited the lowest daily recovery growth rate (0%), followed by Hohhot (2.6%), Xining (5.1%), and Urumqi (7.3%), etc.,

Subsequently, the human combined with natural response dominated the COVID‐19 trajectory concerning recovery after 13 February 2020. The observed trajectory during the valid human response time is shown in Figure 7‐right, wherein except for Hohhot exhibited the increase (+0.8%) in daily recovery growth rate and Lhasa exhibited the steady (0%), other 29 cities exhibited the decrease. However, supposing only the natural response dominates the virus spread during the valid human response time, the predicted contemporaneous trajectory is shown in Figure 7‐down, wherein 10 cities exhibited the increase in daily recovery growth rate, while 21 cities showed the decrease. Specifically, Harbin increased 56.7%, followed by Fuzhou (+6.8%), Nanning (+6.1%), and Hohhot (+4.6%), etc., On the contrary, Zhengzhou decreased 8.8%, followed by Yinchuan (−8.3%), Beijing (−8.2%), and Changchun (−7.3%), etc.,

## 4 Discussion

### 4.1 Natural Attribution of COVID‐19 Trajectory

Those investigated contradictory public researches (Table S5) showed that the possibility still cannot be ruled out that the decrease or increase in COVID‐19 pandemic is partially attributable to other unknown climatic factors. Correspondingly, our research could be, to some extent, a trade‐off for those contradictory conclusions, because we combined all investigated parameters into a single compound model to consider their coupling relationship for more reasonable and logical conclusions about the climatic influence on the COVID‐19 pandemic. In this regard, these researchers could be a significant support for the significance and authenticity of the presented study.

In this study, we synthesized the seven SNF models based on the EDBF strategy, which explicitly evaluated the performance of individual models in simulating observed COVID‐19 trajectory, assigned weights for the models accordingly, and then attributed the contributions of the potential driving factors to spatial COVID‐19 trajectory based on the optimized integration of the seven SNF models. During COVID‐19 transmission, aerosol was assigned the maximum EDBF weight (54.8%), followed by visibility (42.9%), which uncovers the airborne feature of COVID‐19 spread (Sima, 2020; Wang & Du, 2020), to some extent. It is mentioned that aerosol (*p* = 0.01) and visibility (*p* = 0.026) individually have a significantly negative act on the virus spread. Onward, mean visibility decreased significantly, while mean aerosol increased significantly (*p* < 0.001) during March in 31 cities of China (Figure S5‐i). During the complex response to COVID‐19 transmission, the shift of aerosol hinders the virus spread further, whereas visibility promotes it. It is certain that many other natural factors neglected in our work also response to the COVID‐19 trajectory, thus it is essential to consider the interactions between different SNFs response.

Onward, wind speed was the dominant natural factor over 66.8% for COVID‐19 related death, followed by vegetation (16.9%) and visibility (16.4%). Furthermore, the separate response of wind speed to death is significantly (*p* = 0.043) negative. Although vegetation and visibility exhibited 33.3% of EDBF weight, their separate response to death is yet insignificant (*p* = 0.67; *p* = 0.311). Thus, a well ventilated environment in hospital ward could benefit the decrease of COVID‐19 related death. Nevertheless, Figure S5‐II‐f displayed little discrepancy in wind speed between February and March in 31 cities of China. During the complex natural response to COVID‐19 related death in March, wind speed essentially contributes less due to its minor shift. Despite visibility varies greatly, it is little sensitive to this type of death. In this regard, investigated natural attribution of increased growth (Figure 6) in COVID‐19 related death during March is uncertain.

Furthermore, our study revealed that the variety of humidity and barometric pressure dominate COVID‐19 related recovery. Humidity contributed 47.2% of the interaction among compound natural factor to the COVID‐19 response, followed by barometric pressure (34.2%). Moreover, the separate response of humidity and barometric pressure to recovery are both significantly positive (*p* < 0.001). However, the compound response of investigated natural factors resulted in the slight increase in recovery during March (Figure 7), which is the only response of just weather change, and the reason will be discussed in Section 4.2. Although separate humidity during February was well matched with COVID‐19 related recovery (*r* = 0.724), its decrease trend (Figure S5‐III‐g) in March was unlikely to determine the coextensive decrease in recovery. In this regard, the well correlated natural factors with COVID‐19 trajectory reported in previous studies (Sima, 2020; Yoo, et al., 2020) is uncertain to predict the future trajectory due to the complex interaction among natural factors.

### 4.2 Hypothesis of Separation Model

**Figure 8:**
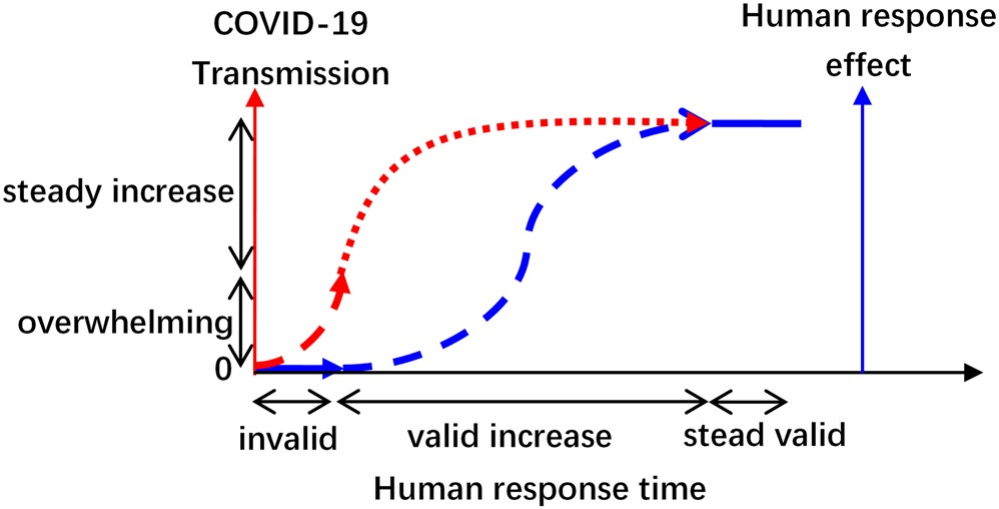
The delayed effect of the human intervention to the COVID‐19 transmission. The blue line and red line represent the human response effect and the COVID‐19 growth curve, respectively. Moreover, the first solid blue line means that it is invalid for curbing the COVID‐19 transmission during the corresponding range of human response time due to the overwhelming COVID‐19 pressure at the beginning of the human response (exponential COVID‐19 growth). As the response time increases, the validity of human response effect to the COVID‐19 trajectory also increases. Thus, the dotted blue line means the increasing valid time of the human response, during which the COVID‐19 has a steady growth (red dotted line).

Due to the average 14 days of COVID‐19 incubation period, both natural and non‐natural factors will certainly have lag effects on the observed COVID‐19 trajectory concerning infection, death, and recovery. To handle this problem, the hypothesis was proposed that there is delayed response of trajectory to non‐natural factors during January 22 to February 12, 2020, that is pharmaceutical (PIs) and non‐pharmaceutical interventions (NPIs). Apart from the COVID‐19 incubation period, the second delayed effect is the weak non‐natural interventions at the beginning of the outbreak. During this period, the human response to the overwhelming COVID‐19 transmission is invalid (Figure 8). In this regard, the COVID‐19 trajectory at the initial time of human response was primarily driven naturally. Therefore, we advanced the reported data ahead for about 23 days to compensate the lag effects discussed above, containing 14 days of COVID‐19 incubation period and 9 days of invalid human response time. However, although Tian investigated the role of human response in curbing the outbreak across China in SCIENCE (Tian, et al., 2020), we do not know the exact time when the human response took effect. It is worthy to mention Figure 1 in the reference (Tian, et al., 2020) which provides the dates of discovery of COVID‐19 and of the human response from December 31, 2019. The novel coronavirus was detected in Wuhan city on December 31, 2019, but the earliest human response was on January 21, 2020. Thus the virus spread naturally for at least 22 days. Moreover, up to February 10, 2020, the closed management was just put in the residential districts in Hubei province. Based on the delayed effect, 7–14 days were invalid response time during the 22 days of human response. In addition, human response has little validity to symptomless cases and cases under the incubation period (14 days). Also, due to the limitation of detection policy and capacity, lots of infected cases cannot be reflected in the reported data. Based on this assumption, the reported data during January 22 to February 12, 2020 approximately uncovers the unreported COVID‐19 trajectory 23 days ago (from December 31, 2019 to January 21, 2020), during which the virus spread naturally (Table S3). We defined this timestamp as invalid human response time. After February 13, 2020, we thought the human response to COVID‐19 trajectory becomes increasingly valid, during which the virus spread under the control of human response. Therefore, the model developed between compound natural factor and COVID‐19 trajectory during the invalid human response time could predict the future trajectory driven by only natural factors. Onward, the model is capable to separate the acting of natural and non‐natural factors on COVID‐19 transmission, which will be discussed in Section 4.3.

### 4.3 Effect Separation of Natural and Non‐natural Factors to COVID‐19 Trajectory

**Figure 9:**
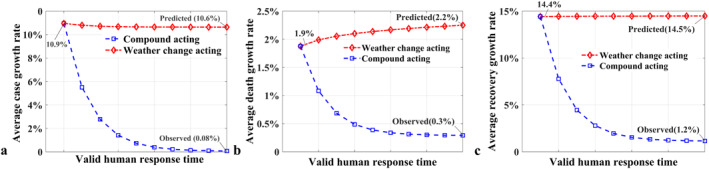
Effect separation (a) Effects of natural factors (−0.3%) and non‐natural factors (−10.5%) to average case growth rate (b) Effects of natural factors (+0.3%) and non‐natural factors (−1.9%) to average death growth rate (c) Effects of natural factors (+0.1%) and non‐natural factors (−13.3%) to average recovery growth rate. Assume compound acting contains natural and non‐natural factors.

Section 4.2 combined with Section 3.3–3.5 supported that the response of COVID‐19 trajectory to natural factors and non‐natural factors is capable to be approximately separated. During the invalid human response time, the average daily case growth rate among 31 cities of China was 10.9% (Figure 9a). Subsequently, after the response of COVID‐19 transmission to human and natural interactions, the observed recovery growth rate decreased about 10.8% (to 0.08%). However, supposing COVID‐19 went on spreading naturally during the valid human response time (Table S3), the growth rate was predicted to decrease only 0.3% (to 10.6%). In this regard, the average decrease of 0.3% in COVID‐19 transmission revealed the interaction outcome of compound natural factor to COVID‐19 trajectory concerning infection. Also, the average decrease of 10.8% illustrated the interaction outcome of both human and natural factors to trajectory. Subsequently, the effect of human (non‐natural) intervention to COVID‐19 transmission is separated, contributing the decrease of 10.5%. The Paired *t*‐test (Table S4) (Rosner, 1982) shows that the effect of non‐natural response to the COVID‐19 virus spread is significant (*t* = −12.919, *p* < 0.001), contributing the decrease of daily case growth rate in all investigated cities of China (mean = −10.9%). In contrast, the effect of natural interaction to the virus spread is insignificant (*t* = 0.019, *p* = 0.985) and region specific, wherein the daily case growth rate in 13 cities (Harbin‐excluded) decreased slightly (mean = −2.6%) while other 16 cities (Lhasa‐excluded) increased (mean = +3.2%) (Figure 5). Specifically, Harbin exhibited the significant decrease (−38%) in daily case growth rate (Figure S7‐a), while Lhasa increased about 11%, because the compound natural factor shifted obviously in Harbin (+87.8%) and Lhasa (−17.5%) during the period (Figure S6‐a). However, in 31 investigated cities, the average shift of compound natural factor is slight (−4.5%). Thus, the effect of decreased natural interaction outcome to subsequent COVID‐19 transmission is positive.

Prior to the valid human response to COVID‐19 transmission, the virus spread naturally and the average daily death growth rate in 31 investigated cities of China was 1.9% (Figure 9b). Onward, the human response to COVID‐19 became valid, the virus went on spreading under the control of human intervention. During the valid human response time, the average daily death growth rate decreased about 1.6% (to 0.3%), which revealed the interaction outcome of both human and natural factors to COVID‐19 trajectory concerning death. Specifically, except for the increased daily death growth rate in Xian (+8.3%), nine cities decreased, while other 21 cities constant (Figure S7‐b). For example, Harbin exhibited the decrease of 12.8%, followed by Nanning (−12.3%), Nanchang (−6.9%), Changchun (−6.2%), Zhengzhou (−6.1%), and Wuhan (−3.9%), etc. Comparably, supposing that the contemporaneous non‐natural intervention to COVID‐19 retained invalid and virus went on spreading naturally, the average daily death growth rate in 31 investigated cities was predicted as 2.2%. In this regard, the response of COVID‐19 death to compound natural factor exhibited the average increase of 0.3%, wherein only seven cities decreased while other 24 cities increased. For instance, Wuhan decreased the most (−8.6%), followed by Beijing (−5.9%), Chengdu (−5.5%) and Harbin (−5%), etc. On the contrary, Nanchang exhibited the increase of 5.2%, followed by Yinchuan (+5.1%), Urumqi (+4.4%), and Zhengzhou (+2.4%), etc., Onward, the effect of non‐natural intervention to COVID‐19 trajectory concerning death is separated, contributing the decrease of 1.9% by subtracting +0.3% effect of natural factors (2.2%–1.9%) from −1.6% of compound factors (0.3%–1.9%). Subsequently, the average shift of compound natural factor in 31 investigated cities is slight (−1.6%). Thus, the effect of decreased natural interaction outcome to subsequent COVID‐19 death is negative.

Additionally, the average daily recovery growth rate in 31 investigated cities of China was 14.4% (Figure 9c) prior to the valid human response to COVID‐19 transmission. Onward, the compound effect of natural and non‐natural factors contributed the average decrease of 13.2% in recovery (to 1.2%). Except for Hohhot (+0.8%) and Lhasa (0%), other 29 cities all exhibited the decrease (Figure S7‐c), wherein Guangzhou decreased the most (−22.8%), followed by Shanghai (−21.6%), Chongqing (−20.3%), and Zhengzhou (−20.3%), etc., Based on the separation model, the interaction outcome of natural factors contributed the average increase of 0.1% in daily recovery growth rate (to 14.5%), while non‐natural factors promoted the average decrease of 13.3% (to 1.2%). Concerning the response to natural factors, daily recovery growth rate increased in 10 cities while decreased in other 21 cities. Specifically, Harbin exhibited 56.7% of increase, followed by Fuzhou (+6.7%), and Nanning (+6.1%), etc., On the contrary, Zhengzhou decreased the most (−8.8%), followed by Yinchuan (−8.3%), and Beijing (−8.2%), etc., Furthermore, concerning the response of daily recovery growth rate to non‐natural factors, all investigated cities exhibited the decrease in recovery, wherein Harbin decreased by 68.4%, followed by Nanning (−19%) and Haikou (−18%), etc., Also, the average compound natural factor in 31 investigated cities exhibited a slight increase (+1.5%), thus, the effect of increased natural interaction outcome to subsequent daily recovery growth rate is promotional.

Comparably, the increased natural interaction (outcome of CNF model) potentially reduced the death growth rate and increased the recovery growth rate, but speeded up the virus spread. Contradictorily, to some extent, the decreased natural interaction curbed the virus spread, but increased the death and decreased the recovery. Besides, due to the response of COVID‐19 trajectory to the shift of some natural factors is insensitive, or the variety of natural factors during the certain period is insignificant, the role of natural factors during the pandemic is region and time specific. Onward, combined with the accessorial natural interaction, the non‐natural factors basically dominated the COVID‐19 pandemic trajectory during the valid human response time. However, when the COVID‐19 trajectory entered the retreated phase (e.g., in China, and Australia, etc.,), the effect of natural interaction to subsequent trajectory became important, which is consistent with the CCTV (china central television) news reported on December 21, 2020 (Liu & Liu, 2020).

However, in most public researches (Table S5) for analyzing the climatic influence on the pandemic, the utilized COVID‐19 trajectory (dependent variable) is the interaction outcome of both natural factors (independent variables) and human interventions (independent variables). Thus, the direct correlation analysis between climatic factors and such COVID‐19 trajectory yet cannot reveal the real climatic influence on the pandemic, because the human interventions are the dominant factors and the accessorial climatic influence will certainly be inhibited (ignored) in such correlation analysis. Owing to this, the influence separation of these two types of driving factors is quite important for the better understanding and authenticity of the climatic influence on the pandemic, which is also the main contribution of the presented study.

## 5 Conclusions

Both natural and non‐natural factors have effect on the COVID‐19 trajectory, and separating these two effects is important for the effective response to the pandemic at different phases. Concerning the difficulty of the study that the observed COVID‐19 trajectory is the outcome of complex interaction among potential driving factors, the separation model is developed based on the hypothesis that there is delayed response of trajectory to non‐natural factors during January 22 to February 12, 2020. Thus, the COVID‐19 virus spread naturally during the invalid human response time (phase‐1). During phase‐2, the virus went on spreading under the control of the valid human response. Hereafter, the virus begins to retreat naturally again (phase‐3). In the phase‐1, the CNF model is developed to reveal the response of COVID‐19 trajectory to compound natural factor, and the weight of each single natural factor expresses their coupling relationship. Subsequently, the coupling relationship is iteratively optimized by empirical distribution based framework (EDBF) to be closer to the real response of COVID‐19 trajectory to the interaction among natural factors. Onward the phase‐2, supposing the virus went on spreading naturally, the subsequent COVID‐19 trajectory is predicted through the variety of natural factors shift in the CNF model. However, the observed trajectory exhibits the outcome of compound interaction among both natural and non‐natural factors. In this regard, subtracting the contemporaneous observed trajectory from the predicted trajectory in phase‐2 approximately reveals the separated effect of non‐natural factors to the pandemic. On the contrary, subtracting the observed trajectory in phase‐1 from the predicted trajectory in phase‐2 approximately reveals the separated effect of natural factors to the pandemic. The outcome of separation model exhibits the principal response of COVID‐19 trajectory to non‐natural factors, and subordinate response to natural factors. In this work, the response analysis of COVID‐19 trajectory to the compound natural interactions offers a new perspective on the response of global pandemic trajectory to environmental changes.

However, the study also has two limitations. First, the number of seven natural factors investigated in CNF model is insufficient to reveal the real response of COVID‐19 trajectory to natural complex interaction. In general, the combination of more natural factors certainly output more precise coupling relationship in CNF model. Second, the hypothesis of separation model brings some uncertainty to the separation outcome between natural and non‐natural factors on the COVID‐19 trajectory. In addition, adding some more natural factors, for example, precipitation (Menebo, 2020), solar radiation (Rosario et al., 2020), air quality index (Ahmad et al., 2021), pollutant particles (Zoran et al., 2020), etc., certainly output more precise coupling relationship in CNF model. Apart from extending CNF model to global‐city level in COVID‐19 spread, it can also be employed for other historical epidemic catastrophes, for example, Ebola virus, SARS‐CoV, Spanish Flu, Yersinia pastis, Swine influenza (A(H1N1)), and AIV to generalize the climatic role in the urgent human public health events.

## Conflict of Interest

The authors declare no conflicts of interest. Moreover, all authors emphasize that Taiwan province is a part of the People's Republic of China, and the reason why Taiwan province did not been colored in the map of China (Figures 5–7) is due to the difficulty of Taiwan related data collection.

## Supporting information

Supporting Information S1

## Data Availability

All original and intermediate data for this work are available in the Zenodo repository (Zhengkang ZUO, Lei Yan, Sana, Yiyuan, Fei, Kaiwen, & Hongying (2021). CNF‐Model (Version v1.0.0) [Data set]. Zenodo. http://doi.org/10.5281/zenodo.4563258.)
