# Supplementary material for: Trajectory Simulation and Prediction of COVID‐19 via Compound Natural Factor (CNF) Model in EDBF Algorithm
Source: Earths Future. 2021 Apr 5;9(4):e2020EF001936. doi: 10.1029/2020EF001936 (PMC8250312; doi:10.1029/2020EF001936)
Supplement: Supplementary file 1 — Supporting Information S1 [file EFT2-9-e2020EF001936-s001.pdf]

[Earth's Future]

Supporting Information for

[Trajectory Simulation and Prediction of COVID-19 *via* Compound Natural Factor (CNF) Model in EDBF Algorithm]

[Zhengkang Zuo[1,†], Lei Yan[1,†], Sana Ullah[1], Yiyuan Sun[1], Fei Peng[2], Kaiwen Jiang[1] and Hongying Zhao[1,†]]

[[1] School of Earth and Space Science, Peking University, Beijing, 100871, China

[2] School of Geosciences, University of Edinburgh, Edinburgh, EH8 9JU, UK]

Contents of this file

Figures S1 to S7
Tables S1 to S5

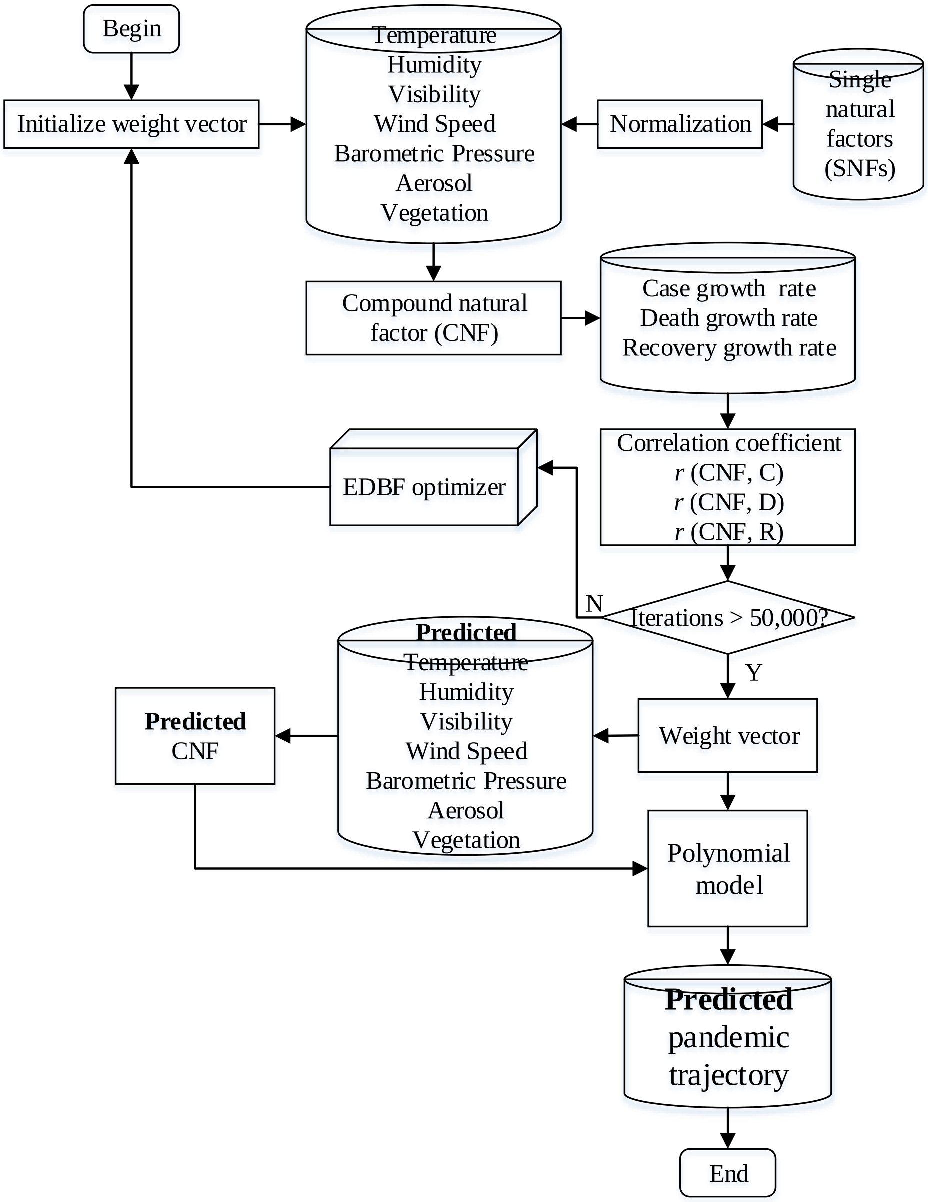

Figure S1. CNF model.

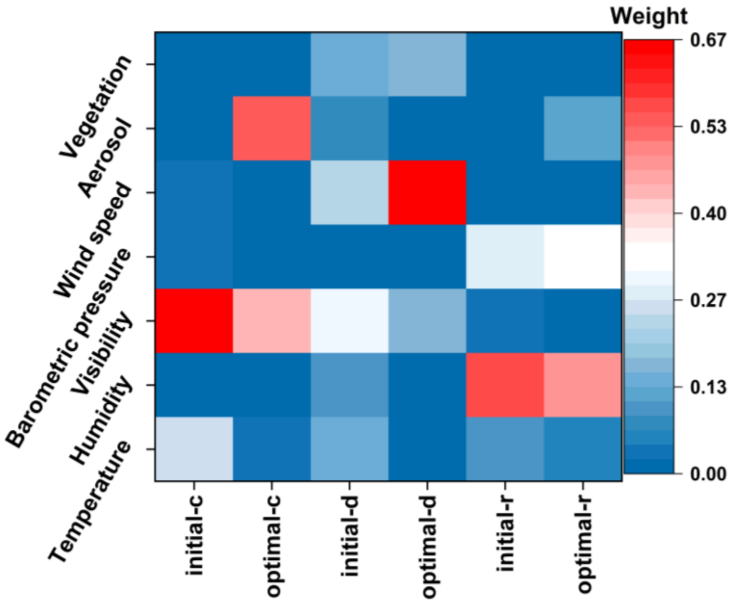

Figure S2. The initial weight values and the optimal weight values in CNF model at each pandemic variable, wherein initial-c and optimal-c correspond to case growth rate, initial-d and optimal-d (death growth rate), initial-r and optimal-r (recovery growth rate).

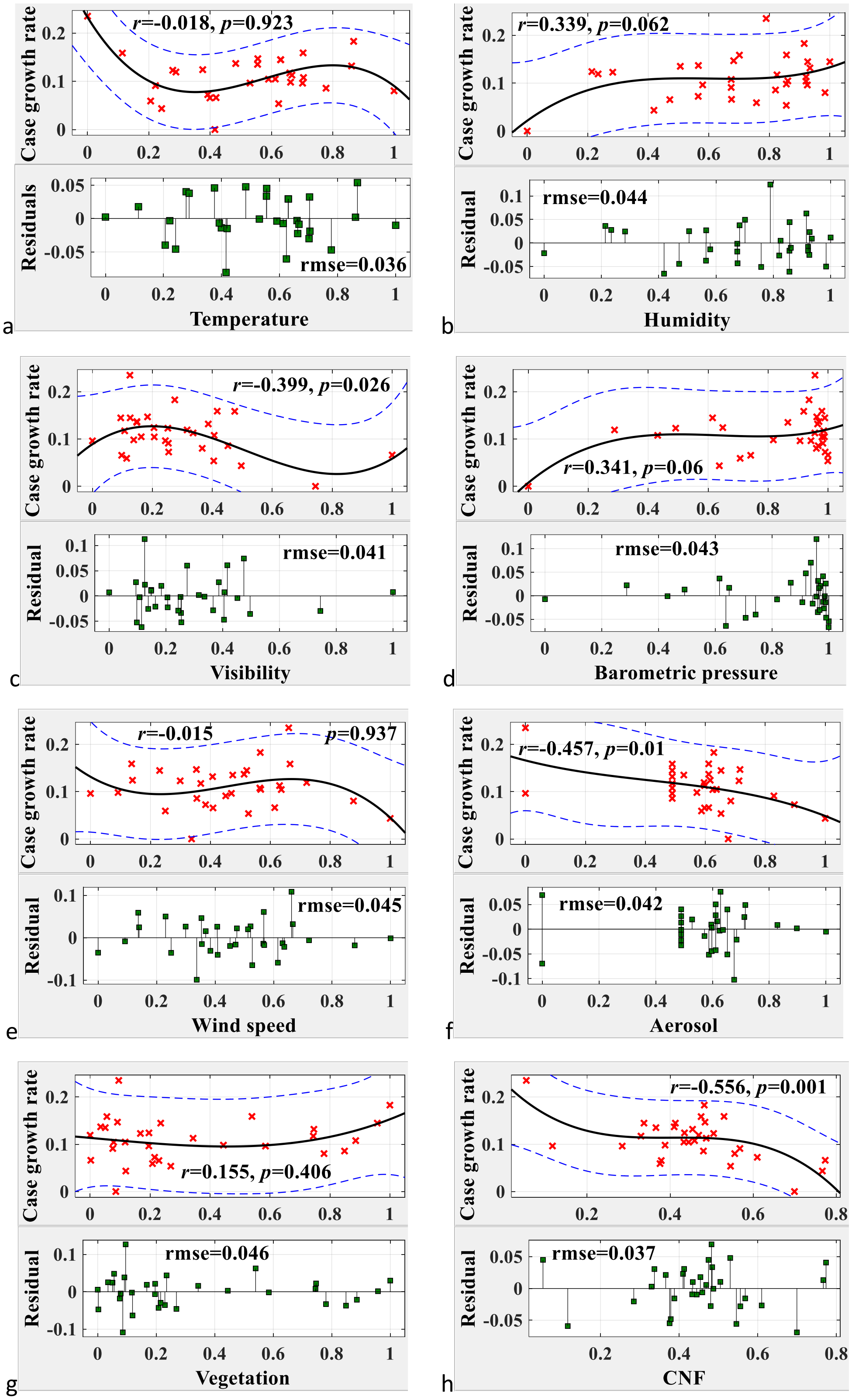

r=-0.018, p=0.923
Case growth rate
Residuals
rmse=0.036
Temperature
a
r=0.339, p=0.062
Residual
rmse=0.044
Humidity
b
r=-0.399, p=0.026
rmse=0.041
Visibility
c
r=0.341, p=0.06
rmse=0.043
Barometric pressure
d
r=-0.015
p=0.937
rmse=0.045
Wind speed
e
r=-0.457, p=0.01
rmse=0.042
Aerosol
f
r=0.155, p=0.406
rmse=0.046
Vegetation
g
r=-0.556, p=0.001
rmse=0.037
CNF
h

(I)

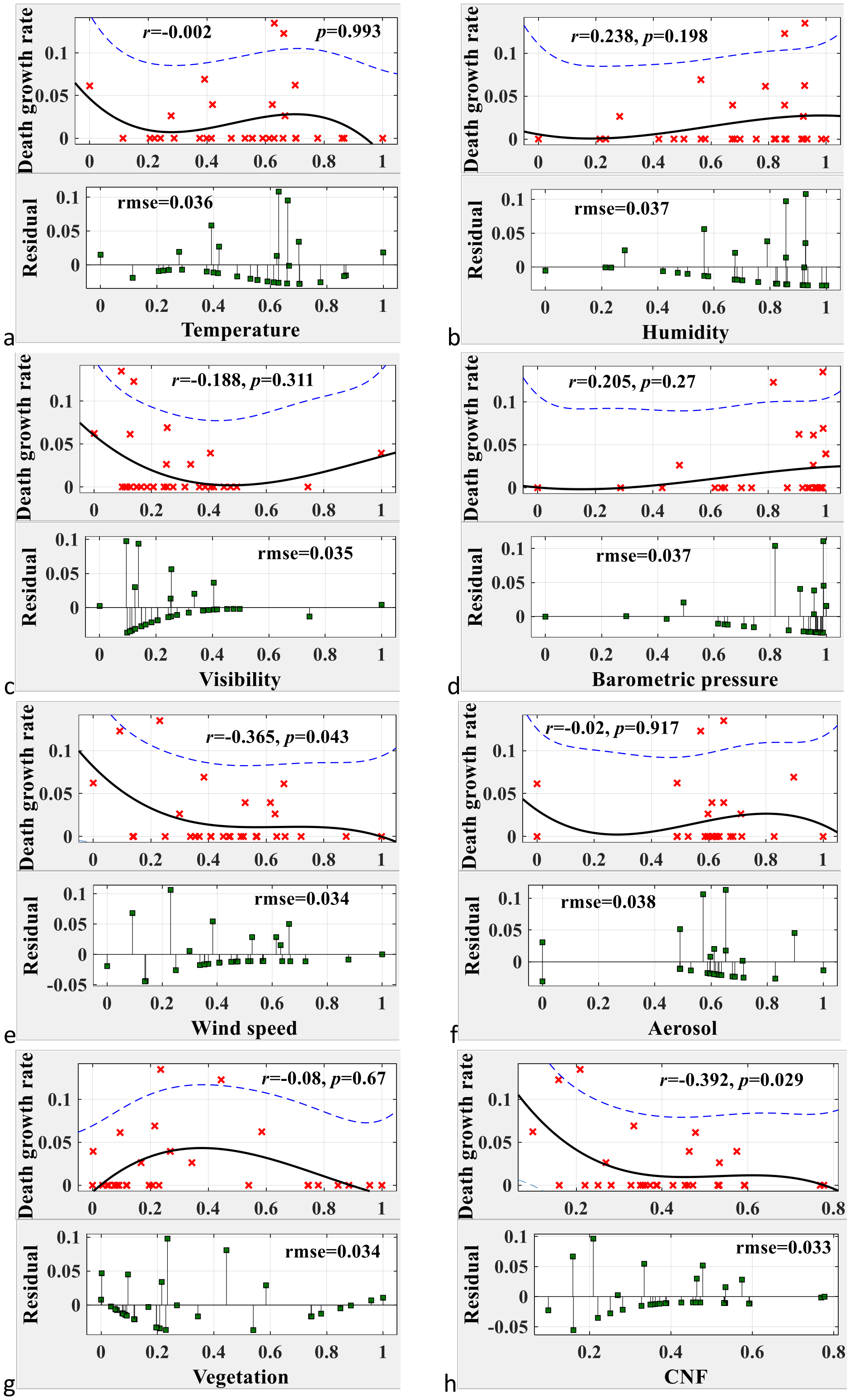

a
Death growth rate
Residual
r=-0.002
p=0.993
rmse=0.036
Temperature
b
r=0.238, p=0.198
rmse=0.037
Humidity
c
r=-0.188, p=0.311
rmse=0.035
Visibility
d
r=0.205, p=0.27
rmse=0.037
Barometric pressure
e
r=-0.365, p=0.043
rmse=0.034
Wind speed
f
r=-0.02, p=0.917
rmse=0.038
Aerosol
g
r=-0.08, p=0.67
rmse=0.034
Vegetation
h
r=-0.392, p=0.029
rmse=0.033
CNF

(II)

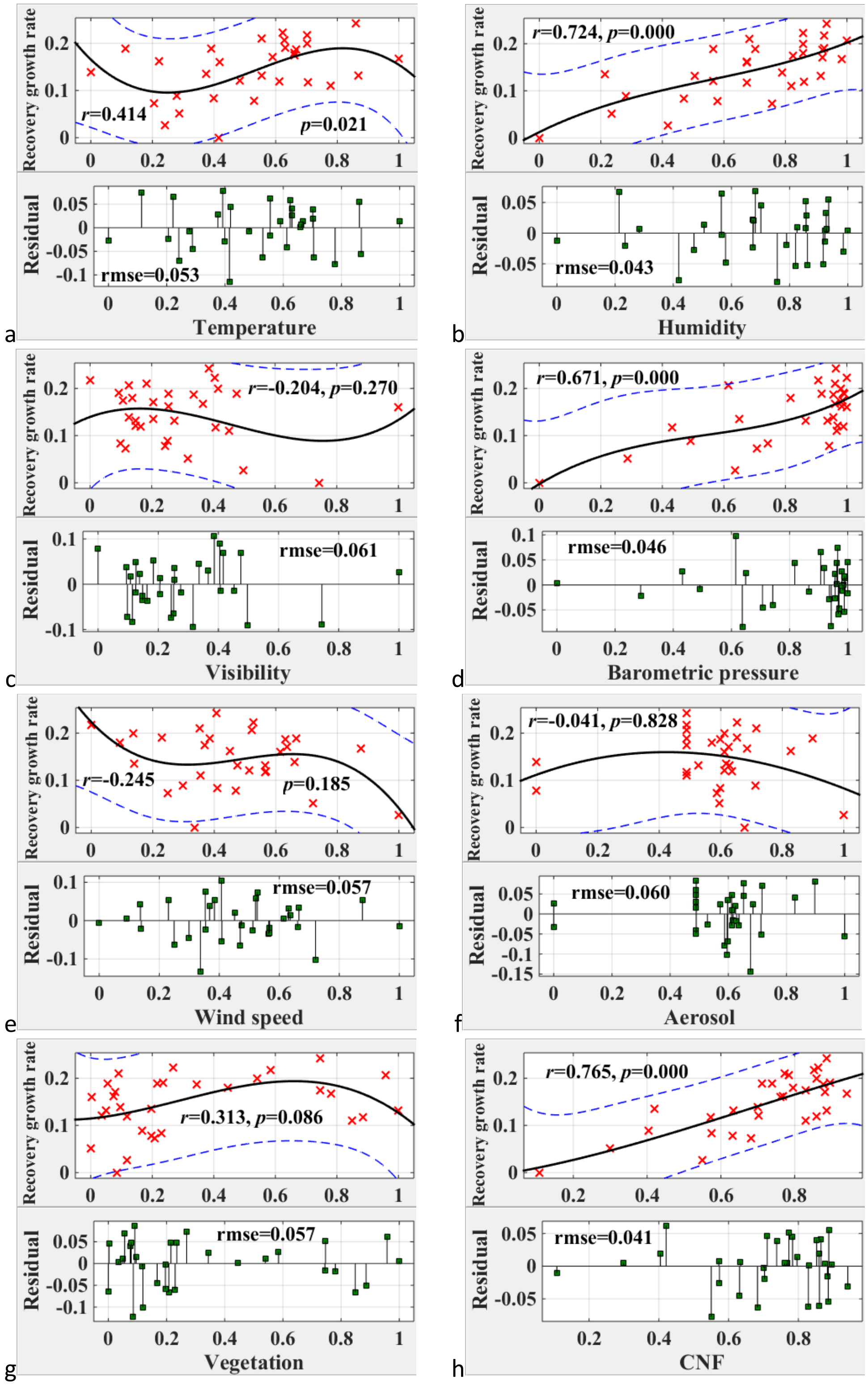

(III)

Figure S3. The relationship between COVID-19 pandemic variables (i.e., daily growth rate in I: case, II: death and III: recovery) and natural factors, i.e., temperature, humidity, visibility, barometric pressure, wind speed, aerosol, vegetation and compound natural factor (CNF).

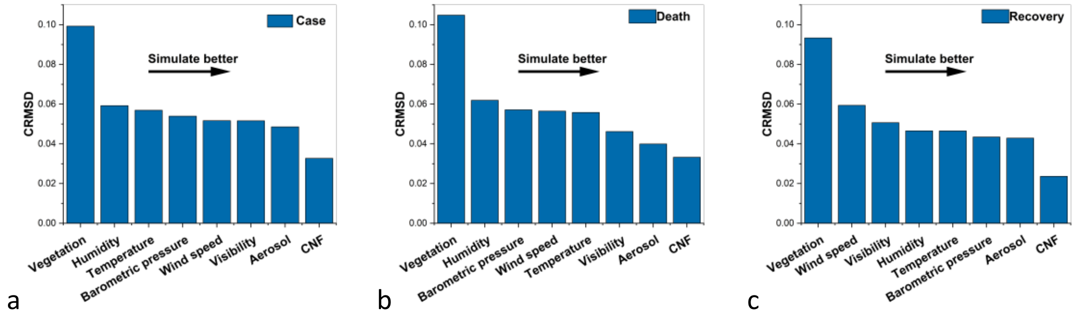

Figure S4. The centre root-mean-square difference between natural factors and COVID-19 trajectory (a) Transmission (b) Death (c) Recovery.

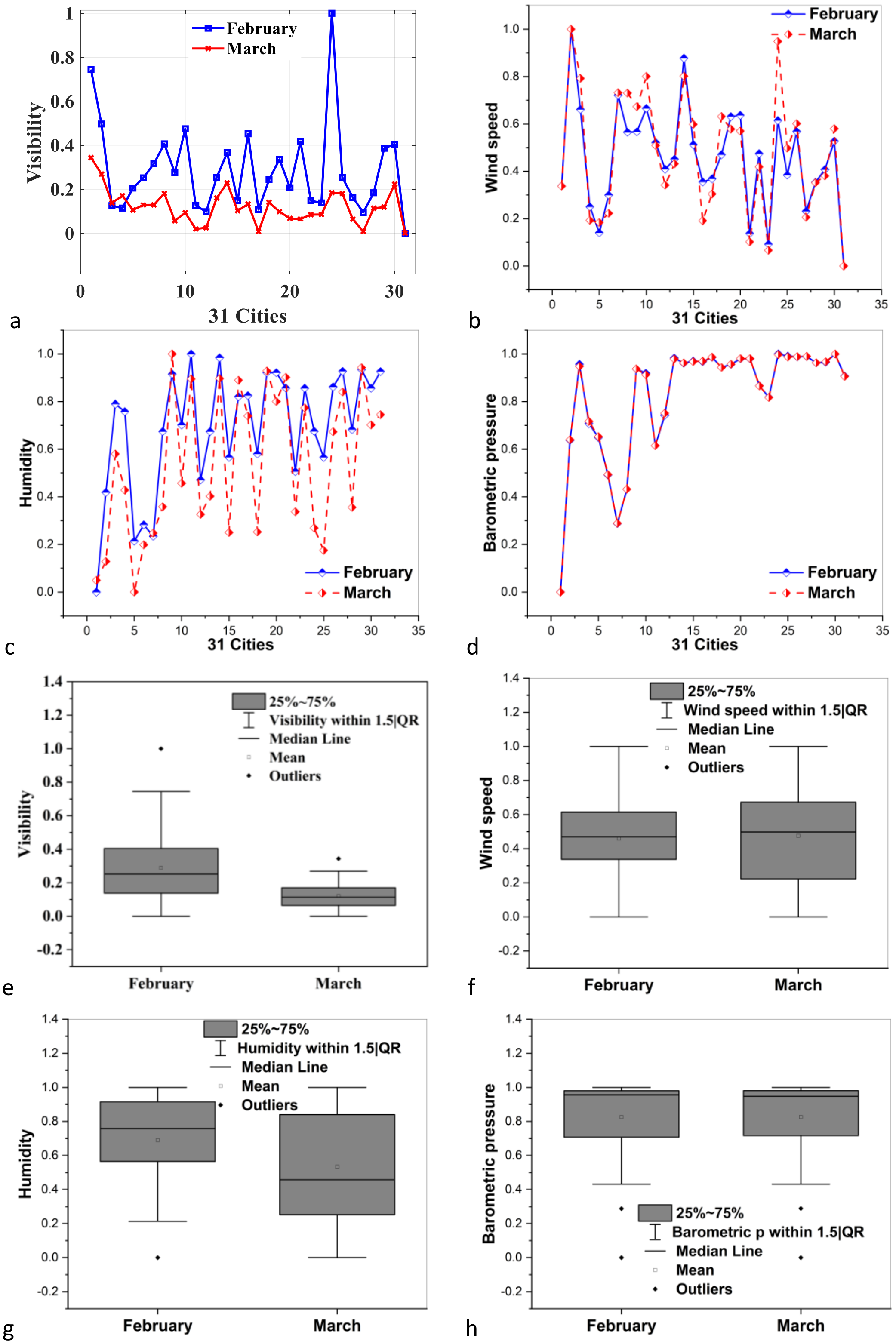

Figure S5. The shift of dominate natural factors which are respond to COVID-19 trajectory during February to March in 31 cities of China. (a, e) Visibility (b, f) Wind speed (c, g) Humidity (d, h) Barometric pressure. The blue line and red line represent city-wise natural factors in February and March, respectively.

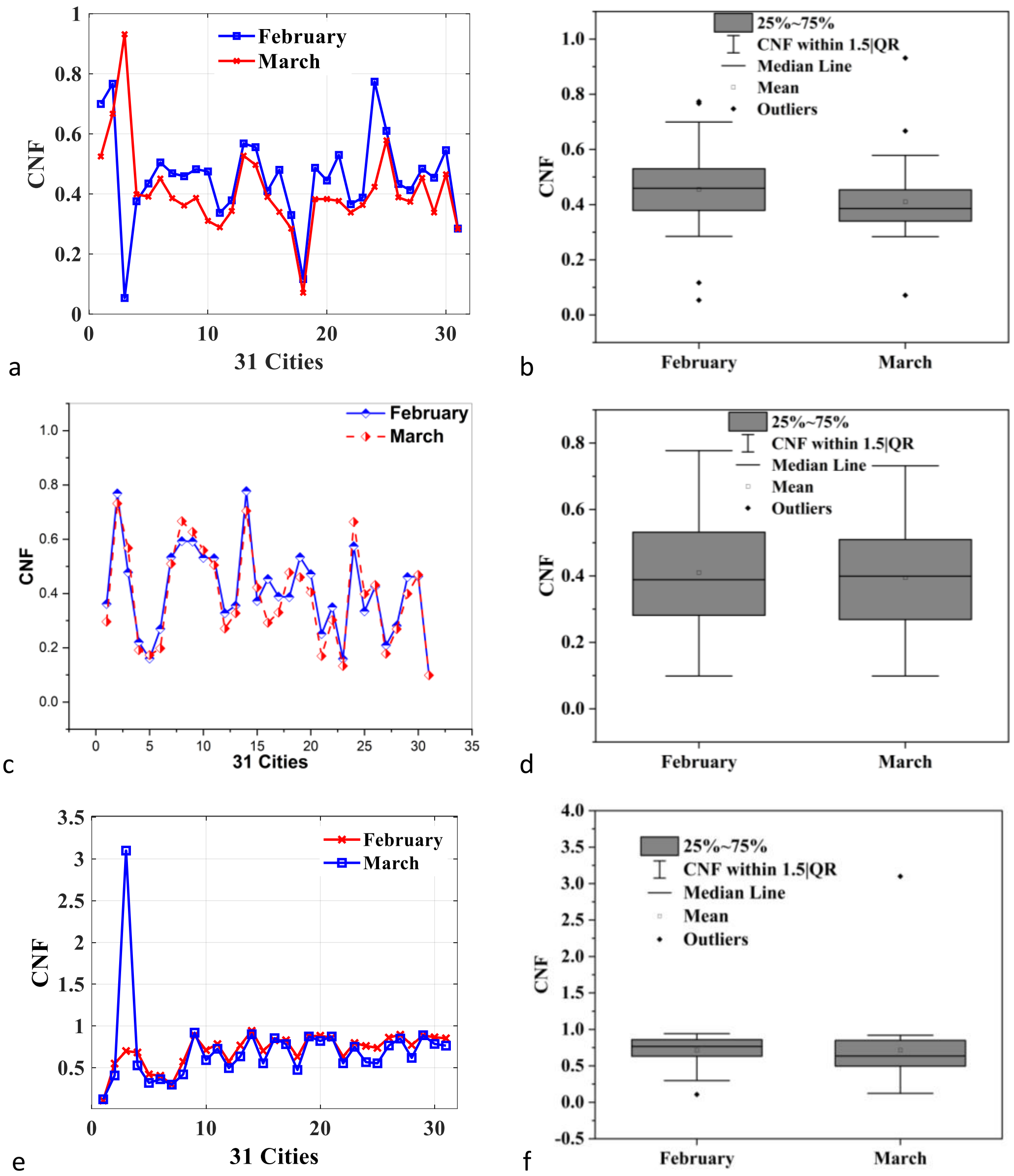

Figure S6. The shift of CNF during February to March in 31 cities of China. (a, d) Case related CNF (b, e) Death related CNF (c, f) Recovery related CNF. The blue line and red line represent city-wise CNF in February and March, respectively.

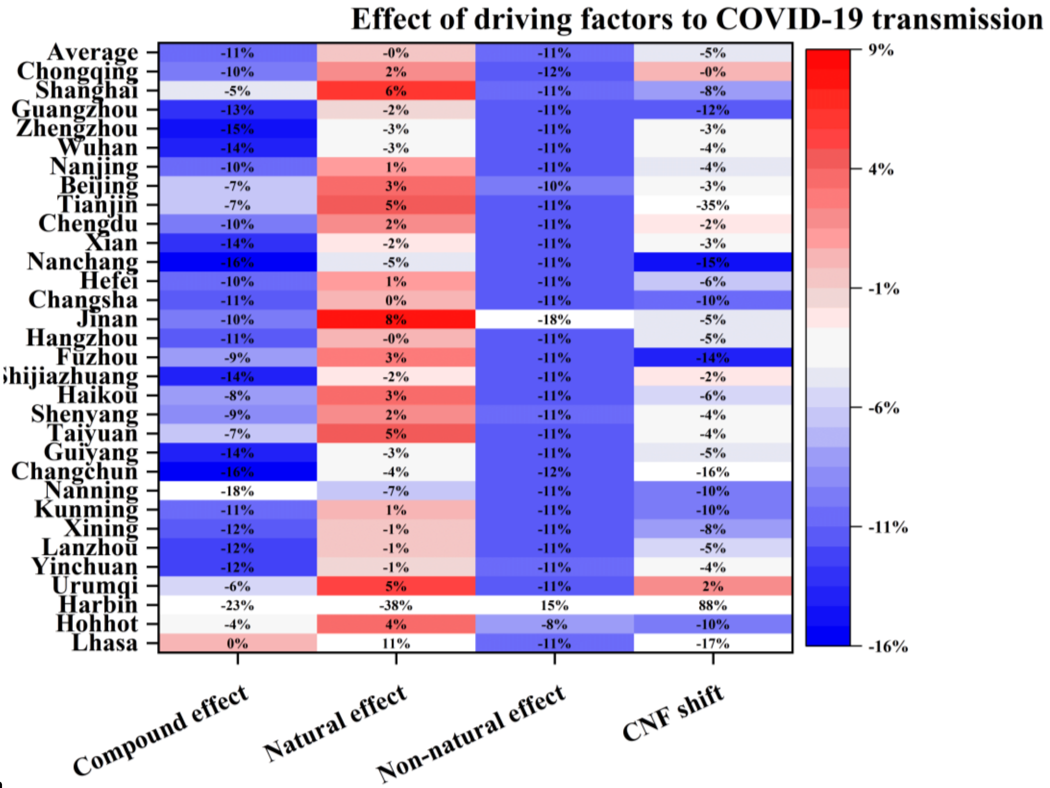
Effect of driving factors to COVID-19 transmission
Average -11% -0% -11% -5%
Chongqing -10% 2% -12% -0%
Shanghai -5% 6% -11% -8%
Guangzhou -13% -2% -11% -12%
Zhengzhou -15% -3% -11% -3%
Wuhan -14% -3% -11% -4%
Nanjing -10% 1% -11% -4%
Beijing -7% 3% -10% -3%
Tianjin -7% 5% -11% -35%
Chengdu -10% 2% -11% -2%
Xian -14% -2% -11% -3%
Nanchang -16% -5% -11% -15%
Hefei -10% 1% -11% -6%
Changsha -11% 0% -11% -10%
Jinan -10% 8% -18% -5%
Hangzhou -11% -0% -11% -5%
Fuzhou -9% 3% -11% -14%
hijiazhuang -14% -2% -11% -2%
Haikou -8% 3% -11% -6%
Shenyang -9% 2% -11% -4%
Taiyuan -7% 5% -11% -4%
Guiyang -14% -3% -11% -5%
Changchun -16% -4% -12% -16%
Nanning -18% -7% -11% -10%
Kunming -11% 1% -11% -10%
Xining -12% -1% -11% -8%
Lanzhou -12% -1% -11% -5%
Yinchuan -12% -1% -11% -4%
Urumqi -6% 5% -11% 2%
Harbin -23% -38% 15% 88%
Hohhot -4% 4% -8% -10%
Lhasa 0% 11% -11% -17%
Compound effect
Natural effect
Non-natural effect
CNF shift
9%
4%
-1%
-6%
-11%
-16%

a

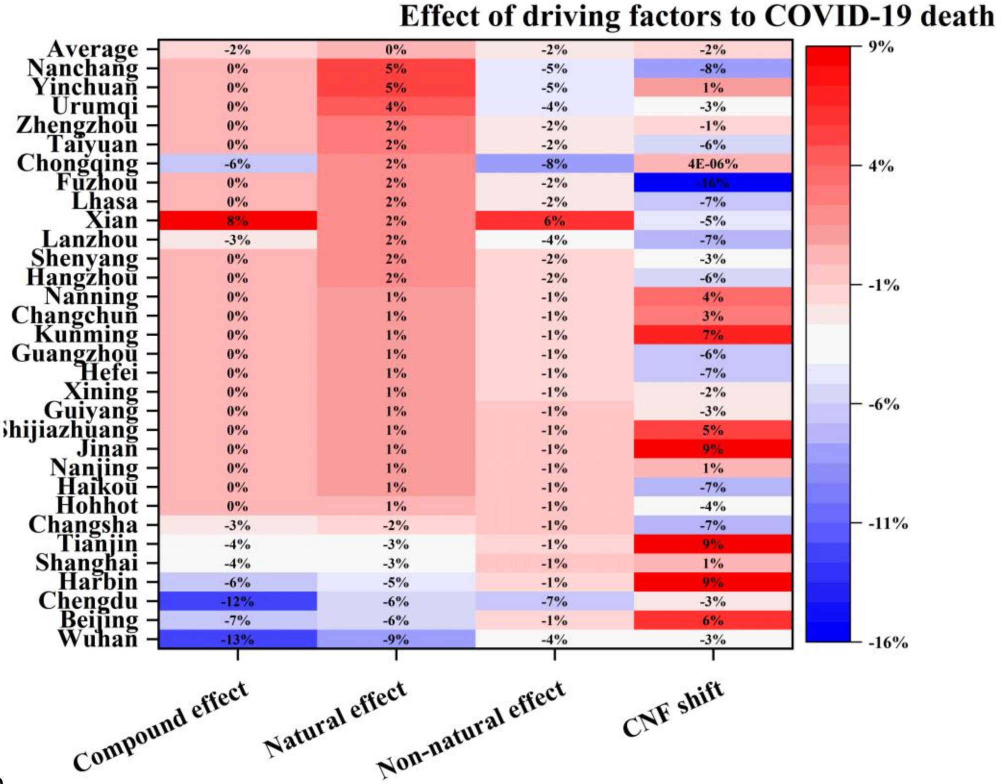
Effect of driving factors to COVID-19 death
Average -2% 0% -2% -2%
Nanchang 0% 5% -5% -8%
Yinchuan 0% 5% -5% 1%
Urumqi 0% 4% -4% -3%
Zhengzhou 0% 2% -2% -1%
Taiyuan 0% 2% -2% -6%
Chongqing -6% 2% -8% 4E-06%
Fuzhou 0% 2% -2% -16%
Lhasa 0% 2% -2% -7%
Xian 8% 2% 6% -5%
Lanzhou -3% 2% -4% -7%
Shenyang 0% 2% -2% -3%
Hangzhou 0% 2% -2% -6%
Nanning 0% 1% -1% 4%
Changchun 0% 1% -1% 3%
Kunming 0% 1% -1% 7%
Guangzhou 0% 1% -1% -6%
Hefei 0% 1% -1% -7%
Xining 0% 1% -1% -2%
Guiyang 0% 1% -1% -3%
hijiazhuang 0% 1% -1% 5%
Jinan 0% 1% -1% 9%
Nanjing 0% 1% -1% 1%
Haikou 0% 1% -1% -7%
Hohhot 0% 1% -1% -4%
Changsha -3% -2% -1% -7%
Tianjin -4% -3% -1% 9%
Shanghai -4% -3% -1% 1%
Harbin -6% -5% -1% 9%
Chengdu -12% -6% -7% -3%
Beijing -7% -6% -1% 6%
Wuhan -13% -9% -4% -3%
Compound effect
Natural effect
Non-natural effect
CNF shift
9%
4%
-1%
-6%
-11%
-16%

b

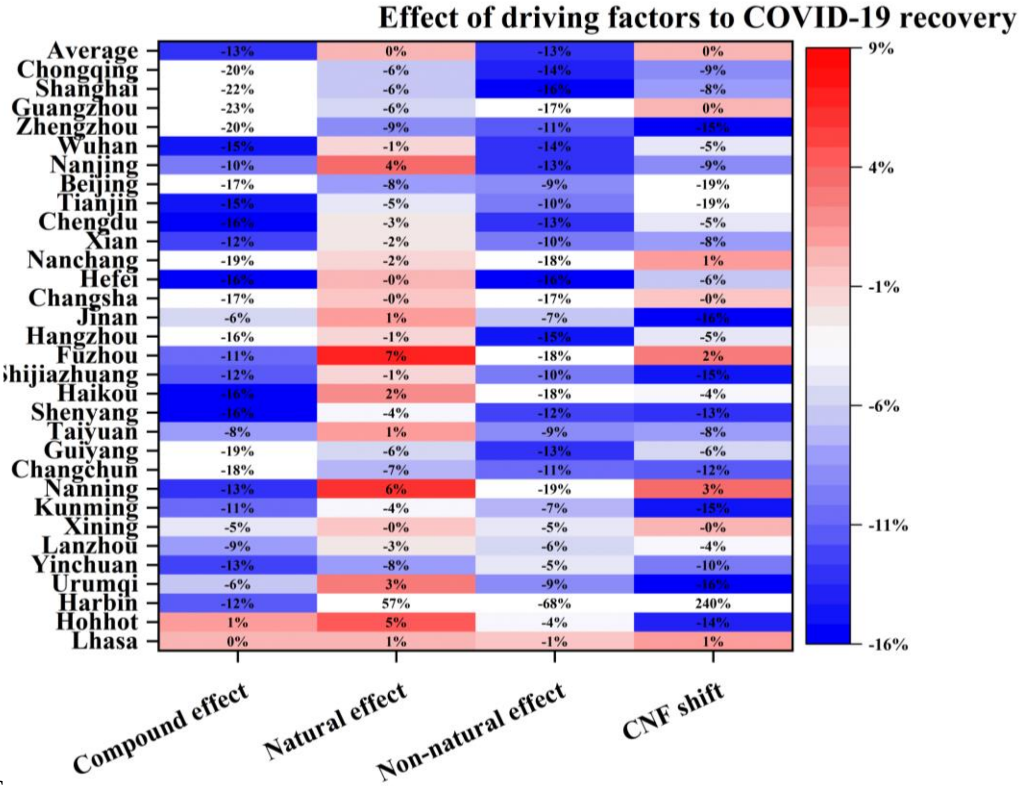

c

Figure S7. Effect of driving factors to COVID-19 trajectory concerning (a) infection (b) death (c) recovery. The first three columns of each figure exhibited the compound effect of natural and non-natural factors, the separated effect of natural factors, and the separated effect of non-natural (human) factors to the COVID-19 trajectory, respectively. It is noteworthy to mention that labels with positive and negative values respectively reveal the positive and negative effects of driving factors to the trajectory.

City name	Data source
Lhasa	http://wjw.xizang.gov.cn/
Hohhot	http://wjw.nmg.gov.cn/
Harbin	http://wsjkw.hlj.gov.cn/
Urumqi	http://wjw.xinjiang.gov.cn/
Yinchuan	http://wsjkw.nx.gov.cn/
Lanzhou	http://wsjk.gansu.gov.cn/
Xining	https://wsjkw.qinghai.gov.cn/
Kunming	http://ynswsjkw.yn.gov.cn/
Nanning	http://wsjkw.gxzf.gov.cn/
Changchun	http://wsjkw.jl.gov.cn/
Guiyang	http://www.gzhfpc.gov.cn/
Taiyuan	http://wjw.shanxi.gov.cn/
Shenyang	http://wsjk.ln.gov.cn/
Haikou	http://wst.hainan.gov.cn/swjw/index.html
Shijiazhuang	http://www.hebwst.gov.cn/
Fuzhou	http://wjw.fujian.gov.cn/
Hangzhou	https://wsjkw.zj.gov.cn/
Jinan	http://wsjkw.shandong.gov.cn/
Changsha	http://wjw.hunan.gov.cn/
Hefei	http://wjw.ah.gov.cn/
Nanchang	http://hc.jiangxi.gov.cn/
Xian	http://sxwjw.shaanxi.gov.cn/
Chengdu	http://wsjkw.sc.gov.cn/
Tianjin	http://wsjk.tj.gov.cn/
Beijing	http://wjw.beijing.gov.cn/
Nanjing	http://wjw.jiangsu.gov.cn/
Wuhan	http://wjw.hubei.gov.cn/
Zhengzhou	http://wsjkw.henan.gov.cn/
Guangzhou	http://wsjkw.gd.gov.cn/
Shanghai	http://wsjkw.sh.gov.cn/
Chongqing	http://wsjkw.cq.gov.cn/

Table S1. City-wise collected data source.

SNF	*r*-value			*p*-value		
	Case	Death	Recovery	Case	Death	Recovery
Temperature	-0.018	-0.002	0.414	0.923	0.993	0.021
Humidity	0.339	0.238	0.724	0.062	0.198	0.000
Visibility	-0.399	-0.188	-0.204	0.026	0.311	0.270
Barometric pressure	0.341	0.205	0.671	0.060	0.270	0.000
Wind speed	-0.015	-0.365	-0.245	0.937	0.043	0.185
Aerosol	-0.457	-0.020	-0.041	0.010	0.917	0.828
Vegetation	0.155	-0.080	0.313	0.406	0.670	0.086

Table S2. Correlation and significance between pandemic variables and single natural factors.

Reported data		Unreported data	
**Invalid human response time**	1-22-2020	**COVID-19 virus spread naturally**	12-31-2019
	1-23		1-1-2020
	1-24		1-2
	1-25		1-3
	1-26		1-4
	1-27		1-5
	1-28		1-6
	1-29		1-7
	1-30		1-8
	1-31		1-9
	2-1		1-10
	2-2		1-11
	2-3		1-12
	2-4		1-13
	2-5		1-14
	2-6		1-15
	2-7		1-16
	2-8		1-17
	2-9		1-18
	2-10		1-19
	2-11		1-20
	2-12		1-21
**Increasing valid human response time**	2-13	**COVID-19 virus spread under the control of human response**	
	2-14		
	2-15		
	2-16		
	2-17		
	2-18		
	2-19		
	2-20		
	2-21		
	2-22		
	2-23		
	2-24		
	2-25		
	2-26		
	2-27		
	2-28		
	2-29		
**Steady human response time**	3-1		
	3-2		
	3-3		
	3-4		
	3-5		
	3-6		
	3-7		
	3-8		
	3-9		
	3-10		
	3-11		
	3-12		
	3-13		
	3-14		
	3-15		
	3-16		
	3-17		
	3-18		

Table S3. The delayed effect of the human intervention to the COVID-19 transmission.

	N	Mean	SD	*t*	*p*
**Natural effect**<br>Predicted CGR(end)-Observed CGR(begin)	32	0.000	0.020	0.019	0.985
**Non-natural effect**<br>Observed CGR(end)-Predicted CGR(end)	32	0.134	0.111	6.810	0.000

Table S4. The significance of natural effect and non-natural effect on the COVID-19 transmission through the Paired *t*-test. CGR (begin) = case growth rate on Janurary-22; CGR (end) = case growth rate on March-18. SD = standard deviation.

References	Temperature	Humidity	WS	Visibility	BP	AOD	FVC
Shi et al, 2020	NA	–	–	–	–	–	–
Notari, 2021	NA	–	–	–	–	–	–
Prata et al, 2020	NA	–	–	–	–	–	–
Xie and Zhu, 2020	PA	–	–	–	–	–	–
Wang, Jiang et al, 2020	NA	–	–	–	–	–	–
Tobías and Molina, 2020	PA	–	–	–	–	–	–
Qi et al, 2020	NA	NA	–	–	–	–	–
Wang, Tang et al, 2020	NA	NA	–	–	–	–	–
Stanam et al, 2020	NOA	–	–	–	–	–	–
Jamil et al, 2020	NOA	–	–	–	–	–	–
Luo et al, 2020	–	NOA	–	–	–	–	–
Ma et al, 2020	PA	NA	–	–	–	–	–
Oliveiros et al, 2020	PA	NA	–	–	–	–	–
Wu et al, 2020	NA	NA	–	–	–	–	–
Mecenas et al, 2020	NOA	NOA	–	–	–	–	–
Haque and Rahman, 2020	NA	NA	–	–	–	–	–
Feng et al, 2020	–	PA	PA	–	–	–	–
Islam et al, 2020	NA	NA	NA	–	–	–	–
Biktasheva, 2020	–	NA	–	–	–	–	–
Sajadi et al, 2020	PA	PA	–	–	–	–	–
Auler et al, 2020	PA	PA	–	–	–	–	–
Şahin, 2020	NA	–	PA	–	–	–	–
Coccia, 2020a	–	–	NA	–	–	–	–
Ahmadi et al, 2020	–	NA	NA	–	–	–	–
Coccia, 2021	–	–	NA	–	–	–	–
Rendana, 2020	NA	NA	NA	–	–	–	–
Rosario et al, 2020	NA	–	NA	–	–	–	–
Coccia, 2020b	–	–	NA	–	–	–	–
Chen et al, 2020	–	–	–	NA	–	–	–
Xu et al, 2020	NA	–	NOA	–	NOA	–	–
Zhu, Liu et al, 2020	NA	NA	NOA	NOA	–	–	–
Srivastava, 2020	NA	NA	–	NA	–	–	–
Paital and Agrawal, 2020	–	–	–	NA	–	–	–
Tosepu et al, 2020	PA	–	–	–	–	–	–
Frontera et al, 2020	–	–	–	NA	–	–	–
Bashir et al, 2020	–	–	–	PA	–	–	–
Martorell et al, 2021	–	–	–	NOA	–	–	–
Lin et al, 2020	NA	–	NA	–	NA	–	–
Zhu, Xie et al, 2020	–	–	–	NA	–	–	–
Breevoort et al, 2020	–	–	–	–	NA	–	–
Semple and Moore, 2020	–	–	–	–	PA	–	–
Takagi et al, 2020	–	–	–	–	NA	–	–
Kumar, 2020a	–	–	–	–	–	PA	–
Kumar, 2020b	–	–	–	–	–	PA	–
You and Pan, 2020	–	–	–	–	–	–	NA
Zhou et al, 2021	–	–	–	–	–	–	NA

Table S5. The comparisons about reported contradictory influences of the investigated climatic parameters on the COVID-19 pandemic. WS = wind speed; BP = barometric pressure; AOD = aerosol optical depth; FVC = fractional vegetation coverage; NA = negative association; PA = positive association; NOA = no association.
